# Supplementary material for: Schistosomiasis and soil-transmitted helminthiasis preventive chemotherapy: Adverse events in children from 2 to 15 years in Bengo province, Angola
Source: PLoS One. 2020 Mar 11;15(3):e0229247. doi: 10.1371/journal.pone.0229247 (PMC7065752; doi:10.1371/journal.pone.0229247)
Supplement: S1 File — (DOCX) [file pone.0229247.s001.docx]

**Schistosomiasis and soil-transmitted helminthiasis preventive chemotherapy: Adverse events in children from 2 to 15 years in Bengo province, Angola - Survey Protocol**

**Purpose**

To analyse the adverse events experienced after the co-administration of Praziquantel and Albendazole as preventive chemotherapy against schistosomiasis and STHs. In addition, to contribute to the knowledge of the adverse effects of anthelmintic medication in Angolan children.

**Study design**

Population-based longitudinal study to survey adverse events in children (2-15 years) at baseline and six month follow up.

**Study area**

The study area is located in Dande, Bengo province, about 60 km from the capital of Luanda. The area is semi-rural with vegetation and water collections and consists of several hamlets that make up CISA (Angola Health Research Centre, translated)'s Demographic Surveillance System (DSS). There are primary and secondary schools where school-aged children attend classes.

**Study population**

The survey will include all children, aged 2 to 15 years in theirs households or in local school, the day after medication with PZQ and ALB.

**Survey timetable**

The area is already mapped by CISA. In December 2012, the children at baseline will provide urine and stool samples for *Schistosoma* and STHs screening and will take a single dose of PZQ e and ALB. The next day, 24 h after medication, children will be surveyed about the adverse events due to the medication (see Standard Operating Procedures in Appendix 1). Six months later, in June 2013, the children will repeat the tests and medication, for the second adverse events survey. In absence of the child on the survey day, the survey taker has to pass the next day (72 h after medication) for recap.

**Sampling method**

Convenient two-stage cluster sampling will be used. The first stage is to sample the villages with the highest prevalence of SCH and STHs. The second stage is to sample a community and a school where children will be picked up for study. All 2 to 15 year olds living in the community and / or studying at the selected school will be invited to participate in the study. The CISA´s DSS has registered over 680 eligible children in these communities.

**Inclusion/exclusion criteria**

All eligible individuals, children aged 2-15, in households or school are to be included. Individuals will be excluded from the study if s/he:

- Has not lived in the study areas for at least six months
- Has a report of a serious adverse event on previous administration of PZQ and ALB.
- Does failed to take PZQ and ALB tablets
- Does not provide informed consent

**Field team**

The field team will be comprised of the following persons:

- One team leader: in charge of coordinating survey activities and ensuring compliance with the mapping protocol
- Two interviewers/ auxiliary workers: to identify households, distribute faecal and urine sample containers, collect household information and refer individuals to a central location to deliver their sample containers for parasitological examination. The day after medication, they return to apply the adverse events questionnaire.
- Three laboratory technicians: one to analyse stool samples, one to analyse urine samples, and one to collect finger-prick blood samples and conduct rapid diagnostic tests (RDT).
- One nurse: to administer PZQ and ALB tablets under direct observation. In addition, AL-20/120mg, on children tested positive for malaria.
- One driver: in charge of vehicle and team safety and security. While the research teams carry out fieldwork, the driver keeps the vehicle and maintains communication between field workers.

The team should be pre-trained in all procedures and conduct a pre-test (pilot study) before starting the study.

**Research Preparations**

Local health and administrative authorities and community leaders will be contacted to obtain permission to conduct the research and to obtain their support and collaboration in planning and conducting the research.

**Therapeutic intervention and AEs survey**

The head of household or guardian of children will be invited to participate in the study. If written consent is given, the child will provide urine, stool and blood samples after a finger prick. Then, all children will receive an intervention kit that comprises a single dose of PZQ (40mg / Kg body weight) and ALB (400mg). AL-20 / 120mg (weight-proportional dose, 3-day schedule) will also be given to children with a positive malaria test, as recommended by the National Malaria Control Program. The next day, 24 to 72 hours after medication, all participants will be surveyed about the adverse effects of the medication. The AEs will be classified as severe if it results in death, hospitalization or prolongation of an existing hospitalization, persistent or significant disability, and accidental or intentional overdose. Otherwise, AEs will be mild or moderate, as recommended by WHO (2016). If some participants are absent at the time of the visit, sample containers will be left with a family member and the next day the field team will pass again. At school, teachers may sign consent when requested or authorized by the child's parents.

**Data and biological samples collections**

A structured questionnaire (translated version in Appendix 2a and original in Appendix 2b) will be used to record all survey data. Each participant will be given a urine specimen container and a stool specimen collection bottle with an indication of the amount of stool required. The stool container will be distributed to individuals on the day before collection. The urine container will be delivered and collected on the day of collection. Parasitological examination of stool and urine samples will be performed in the CISA laboratory. A fingers tick blood sample will also be collected for the rapid malaria test.

.

**Data analysis**

The relevant information to guide the implementation of AEs research, on which we will perform appropriate statistical models, is:

- The percentage of individuals who experienced AEs;
- The symptoms and the intensity of AEs occurred, classifies as milld, moderate and severe;
- The difference between age groups or stratified risk factors, as intensity of infections;
- The predictors of AEs.

**Parasitological methods**

The following information has been adapted from relevant WHO manuals:

- Assuring safety of preventive chemotherapy interventions for the control of neglected tropical diseases [1].
- Basic Laboratory Methods in Medical Parasitology [2].
- Preventive Chemotherapy in Human Helminthiasis: Coordinated Use of Anthelminthic Drugs in Control Interventions [3].
- Helminth control in school-age children [4].

Parasitological diagnosis of schistosomiasis is made by either examining (i) urine (for *S. haematobium*) using the reagent strip for haematuria or (ii) stool samples (for *S. mansoni*) using the Kato-Katz method. However, the study area map only demonstrates the existence of *S. haematobium*.

The reagent strip is a quick and easy method that is also highly sensitive and specific in endemic areas. The reagent strip is dipped into the urine sample and then, after about one minute, compared with a colour scale supplied with the strips. Intensity of infection can be estimated according to the quantity of blood detected by the strip. Since haematuria tends to be more consistent than excretion of eggs, the strips can be used at any time of the day.

One alternative of urine filtration is the examination of the resulting sediment from 10 ml of centrifuged urine and egg counting which also involves microscopic observation and the concentration of the eggs of *S. haematobium*, expressed as eggs/10ml of urine. The urinary excretion of *S. haematobium* eggs follows a daily rhythm, with a peak around noon. Urine specimens form examination are therefore best collected between 10:00 and 14:00 (10 am and 2 pm).

The Kato-Katz technique involves microscopic examination of a fixed amount of faecal material to detect and count *S. mansoni* eggs and STHs eggs and is estimated in eggs per gram of faeces (epg). Epg values are obtained by multiplying the number of eggs counted on the slide by a multiplication factor that varies according to the size of the template used. WHO recommends the use of a template holding 41.7mg of faeces, which corresponds to a multiplication factor of 24. Ideally, all samples should be collected in the morning and processed and examined in the afternoon of the same day.

Capillary blood samples will be collected for malaria diagnosis by rapid diagnostic testing (SD BIOLINE Malaria Ag Pf / Pv, Standard Diagnostics, Inc) and for estimating hemoglobin levels with the Hemocue System (HemoCue® 201+, Angelholm, Sweden).

**Laboratory safety**

Team members will be trained on laboratory safety including wearing of latex gloves during the collection and microscopic examination of faecal and urinary specimens and collection of blood samples. Any material contaminated with stool, urine or blood will be soaked in a suitable disinfectant, such as sodium hypochlorite solution, before disposal or cleaning for reuse. Sharps, such as lancets used to collect finger-prick blood samples, will be collected in safety containers and disposed according to best practice.

**Quality control**

The consistency of microscopic results during the survey should be verified by quality control; this is particularly important for the Kato-Katz technique. Before the survey is undertaken, a day will be spent evaluating the consistency of egg counting among laboratory technicians. Each day during the survey, the team leader will read 10% of the slides handled by each microscopist without prior knowledge of the results. In the case of a discrepancy larger than 10%, the two readers will discuss the slide and further slides will be examined to avoid repeated errors.

**Training**

To perform field research, some experience in diagnostic methods and data collection is required. CISA technicians who participated in the prevalence survey previously conducted in this area will be hired. Other assistants will work alongside these technicians until they have experience in field research methods and practices.

**Ethical considerations**

Study participants will have the purpose of the study explained to them in their local language at the time of recruitment. Written consent will then be requested from heads of household. No payment or other form of compensation will be provided to participants. However, all individuals found to be infected with one or more of the diseases under investigation will be treated with the appropriate anthelminthic drug. If participants want to stop being examined after giving full consent, they are free to do so, and will be excluded from the study.

This study was submitted for approval by the Angolan Ministry of Health (original and translation on appendix 3).

**References**

1. WHO. Assuring safety of preventive chemotherapy interventions for the control of neglected tropical diseases [Internet]. WHO. 2011 [citado 4 de Junho de 2019]. Disponível em: http://www.who.int/lymphatic_filariasis/resources/9789241502191/en/

2. WHO WH. Basic laboratory methods in medical parasitology. World Health Organization; 1991.

3. WHO WH. Preventive Chemotherapy in Human Helminthiasis: Coordinated Use of Anthelminthic Drugs in Control Interventions : a Manual for Health Professionals and Programme Managers. World Health Organization; 2006. 75 p.

4. WHO WH. Helminth control in school-age children: a guide for managers of control programmes. Geneva: World Health Organization; 2011.

**Appendix 1. Standard Operating Procedures**

**Day 1**

1. Sensitization of the population and local authorities

2. Signature of informed consent

3. Distribution of stool containers

**Day 2**

1. With the support of the neighbourhood coordinator, organize local childcare

2. Registration of children

3. Collection of stool container

4. Delivery and collection of urine container

5. Blood sampling and measurement of haemoglobin and TDR for malaria

6. Record data in the general questionnaire

7. Anthropometry

8. Treatment with PZQ, ALB and / or AL

**Day 3**

1. Adverse Effects Questionnaire

2. SCH, STHs egg research in CISA laboratory

**Appendix 2a. Adverse experience reporting form translated**

**Appendix 2b. Adverse experience reporting form original**

**
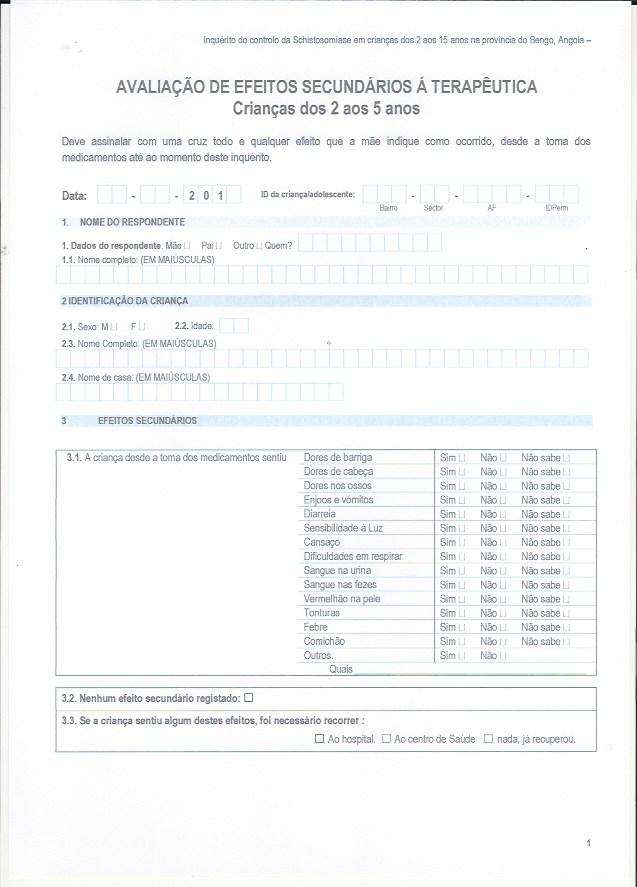
**

**Appendix 3. Adverse experience reporting form**

**From the Ethics Committee of the Angolan Ministry of Health, signed by Dra. Filomena Gomes da Silva, coordinator of the Ethics Committee, in Luanda on December 03^rd^, 2012.**

Opinion on the research protocol entitled "Effectiveness of Schistosomiasis control in children aged 2 to 16 years in Bengo Province, Angola", submitted to this committee by Dr. Miguel Brito, coordinator researcher of the CISA project - Health Research Centre of Angola.

The ethics committee has reviewed the above research protocol and found that it aims to generate information that contributes to better structuring integrated and sustainable control programs for schistosomiasis, intestinal parasites, malaria and anaemia in preschool and school children and in group in high-risk.

Ethical aspects regarding the guarantee of medication and medical care of the study population, obtaining informed consent for participation and the confidentiality of data are safeguarded in the research project. The Committee sees no inconvenience for this study to take place in the country.
