# Supplementary material for: Schistosomiasis and soil-transmitted helminthiasis preventive chemotherapy: Adverse events in children from 2 to 15 years in Bengo province, Angola
Source: PLoS One. 2020 Mar 11;15(3):e0229247. doi: 10.1371/journal.pone.0229247 (PMC7065752; doi:10.1371/journal.pone.0229247)
Supplement: S2 File — (PDF) [file pone.0229247.s002.pdf]

CISA
CENTRO DE INVESTIGAÇÃO EM SAÚDE EM ANGOLA

Efectividade do controlo da Schistosomíase em crianças dos 2 aos 15 anos da província do Bengo, Angola

- Manual de Procedimentos de Campo -

Novembro de 2012

Índice

1. Introdução 4
1.1. Objectivos do Inquérito 4
1.2. Breve Enquadramento Científico 5
1.3. Desenho Geral do Inquérito 7
1.4. Impressos e Documentos de Trabalho 10
1.5. Normas Gerais de Biossegurança 14
1.6. Testes de Diagnóstico rápido – Considerações Gerais 14
2. Organização do Trabalho de Campo 15
2.1. Circuitos de pessoas, amostras e registos 17
2.2. Cronograma-tipo Diário 20
3. Funções e Tarefas por Posição 21
4. Procedimentos Laboratoriais 31
4.1. TDRs na Urina para detecção de Schistossomiase urinária – Trabalhador de Campo na posição 231
4.2. Colheita de sangue capilar – Técnico de Laboratório da posição 2 32
4.3. Procedimento Laboratorial do Paracheck$_{pf}$® - TDR de *Plasmodium falciparum* – Técnico de Laboratório e Trabalhador de Campo na posição 2 35
4.4. HemoCue ® - Determinação da hemoglobina – Técnico de Laboratório da posição 2 38
4.5. Protocolo laboratorial para execução de lâminas para pesquisa de Plasmódio – Técnico de Laboratório da posição 2 .... **Error! Bookmark not defined.**
4.6. Procedimento laboratorial para aplicação da amostra de sangue capilar no papel de filtro .... **Error! Bookmark not defined.**
4.7. Técnica de Kato-Katz - Pesquisa de *Shistosoma* e geo-helmintas – Técnico de Laboratórios na Posição 5 .... **Error! Bookmark not defined.**
4.8. Conservação de amostras de fezes no frasco Parasitrap® - 43
5. Utilização das listagens de agregados familiares e membros seleccionados 45
6. Preenchimento do Questionário 47
7. Questões Comuns e Resolução de Problemas 48

1. Nota Prévia

Neste Manual encontram-se as definições e regras de trabalho necessárias ao trabalho de campo do projeto de investigação "Efectividade do controlo da Schistosomíase em crianças dos 2 aos 16 anos da província do Bengo, Angola".

Espera-se que:

- Leia este manual atentamente e identifique as coisas que não entende e relate-as à equipa de supervisão, seja durante a formação inicial, seja durante o trabalho de campo, de modo a esclarecer a sua dúvida. As regras de trabalho de campo podem ser melhoradas, assim as suas sugestões também serão tidas em conta.
- Tenha o manual à mão quando estiver no campo, habitue-se a consultá-lo sempre que necessitar. Anote os casos em que as suas dúvidas não encontraram resposta no manual e relate-as aos supervisores.

Para ganhar a confiança e a cooperação dos respondentes, a cortesia e a discrição são essenciais. Coordenadores de bairro, *sobas* e outros líderes da comunidade terão de estar sempre informados sobre quem você é, o seu trabalho, a instituição que representa e a fase de trabalho em que se encontra.

2. Introdução

O estudo "Efectividade do controlo da Schistosomíase em crianças dos 2 aos 16 anos da província do Bengo, Angola". adiante designado por "estudo", é um projecto realizado pelo "*Centro de Investigação em Saúde em Angola*" (CISA) sendo parceiro o Ministério da Saúde Angolano (Programa Nacional de Controlo das Doenças Tropicais Negligenciadas e Programa Nacional de Controlo da Malária).

O estudo irá contribuir para a implementação do Plano de Actividades do Projecto CISA em 2009/10, estabelecendo linhas para pesquisas futuras, definindo parâmetros para futuras colaborações, e providenciando um conhecimento aprofundado da extensão destas doenças debilitantes e a necessidade de tratamento.

Os actuais níveis de prevalência da schistossomíase na província do Bengo rondam os 10% em crianças em idade pré-escolar e os 17% em idade escolar, de acordo com o estudo de prevalência realizado pelo CISA em 2010. Estes valores são preocupantes, merecendo um acompanhamento no tratamento destas crianças, de acordo com o proposto pela Organização Mundial de Saúde (OMS). As medidas de controlo estabelecidas pela OMS visam cobrir efectivamente com quimioterapia massiva mais de 75% das crianças afectadas nos Países endémicos. Os serviços de saúde escolar são os mais utilizados, além dos serviços comunitários. Todavia, há controvérsias entre os estudos feitos em diferentes Países quanto ao sistema mais rentável e efectivo de administração massiva do tratamento à população escolar.

2.1. Objectivos do Estudo

Este vai ser um estudo baseado na comunidade e com os seguintes objectivos específicos:

1. Comparar a efectividade do tratamento massivo com praziquantel e albendazol e fornecimento de redes mosquiteiras para crianças em idade escolar, entre um programa escolar e um programa comunitário, em relação à:
   a) Redução da prevalência e severidade da anemia provocada por agentes infecciosos, tais como a malária e a schistosomíase
   b) Redução da prevalência e intensidade das infecções por schistosomíase, parasitoses intestinais e malária

c) Comparar a efectividade entre o tratamento massivo com praziquantel e albendazol e o tratamento selectivo de crianças infectadas para crianças em idade pré-escolar em relação à redução da prevalência e severidade das infecções por schistosomíase e da anemia

2. Caracterizar a adesão ao tratamento, comparando dificuldades encontradas entre grupos etários e programas de distribuição;
3. Determinar a incidência de efeitos secundários durante o tratamento em massa com Praziquantel e Albendazol em crianças em idade pré-escolar, em relação a casos positivos vs casos negativos de schistosomíase e geohelmintíases.
4. Identificar as principais causas genéticas de hemoglobinopatias em crianças dos 2 aos 15 anos, caracterizando as principais alterações genéticas responsáveis por anemia.

2.2. Breve Enquadramento Científico

A schistossomíase, também referida como Bilharzia, e em Angola referida como Sangue na Urina, Samba ou Kussussa, é uma patologia causada por parasitas do género *Schistosoma* e que afecta cerca de 200 milhões de pessoas em todo o mundo. Existem 4 espécies associadas a patologia no Homem: *S. haematobium,* responsável por schistossomíase urinária e *S. mansoni, S intercalatum* e *S. japonicum,* responsáveis por schistossomíase intestinal.

Para o diagnóstico de Schistossomíase vai-se proceder à recolha de amostra de urina e de fezes para a pesquisa de ovos de *S. mansoni* nas fezes e de ovos de *S. haematobium* na urina através da técnica de Kato-Katz. O teste permite ainda a contagem dos ovos, possibilitando estimar a intensidade da infecção. Ais ainda será pesquisada hematúria (sangue na urina) com recurso a fitas para análise de urina (Combur 10 Test, Roche), sendo usado como indicador de infecção por ***S. haematobium***. Além disso, permite quantificar o número de eritrócitos presentes o que é um sinal da intensidade da infecção.

As amostras de fezes e de urina e serão guardadas em tubo eppendorf e posteriormente, no laboratório, será efectuada a pesquisa de *S. mansoni* e *S. haematobium* por t´cnicas de biologia molecular, nomeadamente PCR em Tempo Real.

A malária é endémica no continente Africano, estimando-se que todos os anos seja responsável pela morte de mais de 1 milhão de pessoas em todo o mundo. Existem 4 espécies responsáveis pela infecção: P. *falciparum, P. ovale, P. vivax e P. malariae*. Destes, o *P. falciparum* é o mais prevalente e o responsável por doença mais grave, com maior morbilidade e mortalidade. A detecção precoce

da malária por *P. falciparum* é de extrema importância devido à possibilidade de ocorrer malária cerebral e resistência aos anti-maláricos associadas a esta espécie.

Neste estudo, será efectuado o teste de diagnóstico (TDR) de detecção de antigénio do Plasmódio *falciparum* e das outras espécies não *falciparum* (SD BIOLINE Malaria Antigen Rapid Test series, Standard Diagnostics, Inc.).

A anemia traduz-se por níveis reduzidos de hemoglobina, quando comparados com os valores normais em indivíduos do mesmo género e idade, conforme seguinte orientação:

- Anemia leve: ≥ 10g/dl e menor que o valor normal
- Anemia moderada: 7.0 – 9.9 g/dl
- Anemia grave: <7g/dl

As principais causas de anemia são a má nutrição, associada a deficiência de ferro, folatos ou vitamina A; infecções de origem bacteriana, viral ou parasitária, particularmente devido a malária *falciparum*, parasitoses intestinais, Infecção VIH/SIDA, Leishmaniose, Tuberculose, Trypanosomiase africana e causas genéticas, nomeadamente hemoglobinopatias

A determinação do nível de hemoglobina no presente estudo, será efectuada com recurso ao fotómetro **HemoCue® 201+,** um equipamento leve e portátil que permite determinar a hemoglobina com rapidez, facilidade e com resultados fiáveis. Mais ainda, será recolhida uma amostra desangue em cartão, que permitirá no laboratório a determinação de mutações responsáveis por hemoglobinopatias e pela deficiência da enzima G6PD, responsáveis por anemias graves.

3. Desenho Geral do Estudo

a) Métodos –Localização

A localização do estudo é a mesma do Sistema de Vigilância Demográfico (SVD) que o CISA está a implementar: Caxito, Mabubas e Úcua onde o censo inicial registou uma população estimada de 60.597 indivíduos.

b) Métodos – Amostra da População

O processo-base de amostragem seguido será:

1) Selecção aleatória de **bairros** por comuna do SVD a menos de 5 km de um corpo hídrico de água doce (necessário para potencial existência de schistosomiase).

2) Dentro de cada local de amostragem por bairro, serão inquiridos **agregados familiares** (agregados familiares) escolhidos aleatoriamente da lista de agregados familiares do SVD que preenchem o critério de inclusão [que tenham pelo menos uma criança entre os 2 e os 15 anos de idade. Os agregados familiares serão escolhidos de forma a potenciar um máximo de 80 crianças, e assumindo 50% de recusa/ausência, perfazendo um total de cerca de 40 amostras por local/dia.

3) Nas escolas serão contactados os directores e escolhido um número de turmas que permita incluir cerca de 80 crianças, e assumindo 50% de recusa/impossibilidade dos país se deslocarem á escola, perfazendo um total de cerca de 40 amostras por escola/dia.

c) Métodos – Mobilização da comunidade

Antes de arrancar com o estudo é necessário obter as autorizações necessárias e assegurar que os diferentes níveis da administração e a população estão devidamente informados. Assim é de salientar os seguintes passos:

- Encontro com Administração Municipal e pedido de apoio para o estudo;
- Encontros com os administradores das comunas e dos bairros seleccionados de forma a receberem informação sobre o estudo a ser efectuado e a necessidade da sua participação na sensibilização das famílias escolhidas;

- O Projecto CISA irá informar os coordenadores dos bairros relativamente ao dia do estudo através de telemóveis e rádio, de forma a activar a sensibilização das famílias a serem estudadas (2-3 dias antes da visita para realização do estudo);
- Encontros com os directores das escolas e com os professores e os pais dos alunos.
- O técnico de campo avançado no dia antes do estudo distribuirá panfletos informativos /termos de consentimento e copos para a recolha da amostra de fezes aos agregados familiares seleccionados, ou aos pais que vão á reunião da escola dos filhos.

d) Métodos – Técnicas de Diagnóstico

Aos indivíduos seleccionados irá pedir-se, após serem informados e com o seu consentimento, uma amostra de urina e de fezes, bem como a extracção de sangue por picada no dedo, para as seguintes técnicas:

- **Amostra de urina:**
  - Combur 10 Test, para o diagnóstico de hematúria, que nos dá informação de possível infecção por *S. haematobium*.
  - Filtração de urina para confirmação de diagnóstico de *S. haematobium* no laboratório.
  - Pesquisa de *S. haematobium* por PCR em tempo Real no laboratório.
- **Amostra de fezes:**
  - Diagnósticos de geo-helmintas e schistossomíase intestinal (*S. mansoni*) através de microscopia usando a técnica de Kato Katz em todas as amostras colhidas no campo e a técnica de concentração de fezes no laboratório (em 50% das amostras colhidas).
  - Pesquisa de *S. mansoni* por PCR em tempo Real no laboratório.
- **Amostra de sangue por picada no dedo:**
  - Diagnóstico dos casos *positivos para Plasmodium* usando os testes rápidos SD Bioline.
  - Medições de hemoglobina usando um fotómetro HemoCue® para identificação de casos de anemia.
  - Papel de filtro para posterior extracção de DNA e genotipagem por PCR em Tempo Real de mutações responsáveis por hemoglobinopatias e defenciencia de enzima G&PD..

Mais ainda será efectuada a medição de peso e altura (que no caso das crianças menores de 5 anos vai ser também avaliada pela medição do perímetro braquial a meia altura (PBMA)).
Será ainda aplicado ao ou responsável pela criança/adolescente um questionário que tem como objectivo avaliar os factores de risco local de schistossomíase e parasitoses intestinais, e providenciar uma melhor compreensão das infra-estruturas e cuidados de saúde locais.

e) Métodos – Tratamento

Posterior ao diagnóstico das diferentes situações clínicas, será administrado tratamento da schistossomíase e malária aos casos com resultados positivos nos testes de diagnóstico rápido. Todos os casos que necessitem de acompanhamento posterior serão devidamente encaminhados para as unidades de saúde locais que se encontrarão devidamente informadas da implementação deste estudo. Será ainda entregue 1 mosquiteiro a cada agregado familiar que participar.
No caso de crianças entre os 2 e os 5 anos, será efectuado um questionário pelo Técnico de Campo avançado, no dia seguinte ao estudo, com o objectivo de identificar efeitos secundários do tratamento com Albendazol e Praziquantel.
Mais ainda, para o caso das crianças dos 2 aos 5 anos, serão seleccionados alguns bairros onde o Albendazol e Praziquantel só será distribuído ás crianças com diagnóstico positivo para Schistosomiase, procedendo-se para tal a uma visita dois dias depois da colheita de amostras.

Constituição da Equipa de Trabalho

Equipa de investigadores e de supervisão:

- Investigador Coordenador: Miguel Brito
- Investigador: Manuel Lemos (Médico)
- Investigadora: Chissengo Lucama (Médica)
- Investigador: Clara Mirante (Técnica de Análises Clinicas)
- Investigador: Sofia Moura (Técnica de Análises Clinicas)
- Investigadora Carolina Gasparinho (Enfermeira)

Equipa operacional:

1 coordenador

1 técnico de enfermagem (TE)

1 Técnico de laboratório

3 trabalhadores de campo (TC) (um avançado, um que vai com a equipa para o campo e um para o questionário de efeitos secundários)
2 motoristas

Funções:

- Um Coordenador – coordena todo o trabalho e preenche o questionário
- um técnico de enfermagem - medição de peso e altura e PBM (em crianças com menos de 5 anos), realização do teste das tiras de urina (e avaliação dos resultados) e administração de terapêutica;
- Um técnico de laboratório Execução da punção capilar para testes de diagnóstico rápido da malária, medição da hemoglobina pelo HEMOCUE e papel de filtro.
- Um trabalhador de campo - registo dos presentes que se apresentem com amostra de fezes no dia da amostragem, e iniciar preenchimento do inquérito;
- Um trabalhador de campo avançado – contacto dos agregados familiares e distribuição de frascos para fezes e termos de consentimento no dia anterior ao inquérito,) (2ª, 3ª e 4ª feira)
- Trabalhador de campo 3 - Realizar inquérito de efeitos secundários no dia posterior á amostragem de campo nas crianças dos 2 a 5 anos (4ª, 5ª e 6ª feira)
- Dois Motoristas – transporte e auxilio na recepção dos participantes e no transporte dos técnicos de campo.

A cada grupo corresponde uma definição de termos de referência sem prejuízo das definidas individualmente para cada posição de trabalho e necessidades pontuais do trabalho.

Adicionalmente existirão **2** técnico de laboratório na sede do Projecto CISA, realização da técnica de concentração das fezes e filtração da urina e pesquisa de ovos de *Schistosoma spp*.

4. Impressos e Documentos de Trabalho

Sendo este um inquérito assente na recolha de dados torna-se parte fundamental do trabalho de todos, o conhecimento e reconhecimento da utilidade e funções de cada um dos impressos/formulários/documentos de apoio, sendo enumerados de seguida, quais os existentes e a sua função:

1. Esquema de logística de campo (e funções).

2. Listagens de agregados familiares e membros seleccionados: para o Trabalhador de Campo avançado utilizar no dia de entrega de TdC e frascos para fezes, assim como para auxiliar o trabalho do Trabalhador de Campo móvel e fixo nos dias do inquérito.
3. Folheto informativo e Termo de consentimento: para o Trabalhador de Campo avançado entregar juntamente com o frasco de recolha de fezes
4. Guia para colheita de amostra de fezes: para entregar aos agregados familiares seleccionados juntamente com os frascos de recolha de fezes
5. Questionário: documento transversal a todo o inquérito, que reúne toda a informação não-clínica e parte dos dados clínicos dos inquiridos, deve ser entregue ao cuidador da criança/adolescente na posição 1 que ficará responsável por ele até ao fim do circuito.
6. Questionário de efeitos secundários para crianças dos 2 aos 5 anos.
7. Imagens de eczemas provocados por parasitas intestinais para auxílio dos membros do agregado familiar inquiridos na resposta à questão nº 7 do quadro 5.
8. Impresso de prova de tratamento e diagnóstico: documento a entregar a todos os inquiridos pelo técnico de enfermagem, que reúne os seus resultados de diagnóstico e medicamentos administrados.
9. Impresso de encaminhamento para casos de anemia: documento a entregar a todos os casos com anemia (Hb inferior a 9,9 g/dl) pelo técnico de enfermagem, para posterior informação do pessoal da unidade de saúde mais próxima.
10. Impresso de encaminhamento para casos de malnutrição: documento a entregar a todos os casos de malnutrição (P/A<70% ou/e PBMA<13,5 cm) pelo técnico de enfermagem, para posterior informação do pessoal da unidade de saúde mais próxima.
11. Listas de controlo de material: para utilização diária da equipa, como instrumento de apoio de modo a certificar que todo o material necessário está pronto a ser transportado e usado.
12. Manual de campo: para que possa consultar caso tenha dúvidas ao longo do dia de trabalho.
13. Tabelas de peso/altura: para o pelo técnico de enfermagem determinar os casos em que P/A<70% (só para menores de 5 anos).
14. Tabela para administração de medicamentos

Todos estes documentos encontram-se em anexo a este manual, sendo que as regras específicas para o seu preenchimento encontram-se expressas na definição de tarefas em cada posição no capítulo 3.

a) Identificação dos inquiridos

Os formulários e o questionário possuem em comum a identificação do agregado familiar e/ou do membro desse agregado que está a ser inquirido. Este processo de identificação de agregados familiares e seus membros assenta no processo criado pelo SVD, sendo assim importante definir os seguintes conceitos:

- **Agregado familiar:** Um agregado familiar é um grupo de duas ou mais pessoas, que podem ser ou não da mesma família, que geralmente vivem na mesma habitação e são dependentes entre si.
- **Membro do agregado familiar inquirido:** é toda a pessoa que resida no agregado familiar considerado e previamente seleccionada de forma aleatória.

A cada agregado familiar foi atribuído um número de identificação à sua habitação familiar (isto é, toda e qualquer casa, parte de casa ou grupo de casas que corresponda ao alojamento de um agregado familiar). Na realidade, o que foi numerado é um agregado familiar, mas a placa – preta com letras brancas (ver imagem abaixo) - tem de ser fixada na parede da casa ou de uma das construções que compõem a habitação familiar.

O número de identificação de agregado familiar tem 8 caracteres e é composto por:

- **Sigla do Bairro:** 3 letras.
- **Código do sector:** 2 dígitos.
- **Número sequencial do agregado familiar dentro do sector:** 3 dígitos.

KJM 02
015

Com base no exemplo da placa acima expresso, o preenchimento correcto da identificação do agregado familiar é:

ID do AF: K J M - 0 2 - 0 1 5

Bairro		Secto r		AF
K J M	-	0 2	-	0 1 5

Como se vê, uma ID do AF (identificação do agregado familiar) corresponde a uma localização. Adicionalmente é atribuído uma **ID individual permanente,** que fica assim definida como sendo um código com 11 caracteres, que identifica cada indivíduo, composto pela identificação do agregado familiar e um número sequencial entre as pessoas que fazem parte do agregado familiar (3 dígitos por ordem sequencial do chefe do agregado para os restantes membros).

Se assumirmos que o membro inquirido possui como número de membro o 003, temos que o preenchimento correcto da sua identificação nos formulários e inquérito é:

**ID individual permanente**	K	J	M	-	0	2	-	0	1	5	-	0	0	3
	Bairro				Sector			AF				Membro		

Neste caso, KJM-02-015-003 significa que esta pessoa, reside no bairro KiJoãoMendes, sector 2, casa 015 e foi recenseada em 3º lugar dentro do agregado familiar.

É muito importante que se transcreva sempre esta identificação correctamente, em TODOS os impressos, uma vez que um erro será desastroso para o inquérito.

4.1. Normas Gerais de Biossegurança

Constituindo uma parte significativa do trabalho a realizar diariamente, todos os trabalhadores e supervisores envolvidos no campo ou no laboratório deverão recordar **normas gerais de segurança biológica**, de modo a protegerem-se e a proteger os outros. A saber:

1. O pessoal envolvido no Inquérito deve utilizar luvas em todos os trabalhos que impliquem contacto directo com as amostras biológicas (sangue, fezes ou urina) e manter as precauções normais durante todo o processo de manuseamento e destruição das amostras.
2. Após utilização, devem tirar-se as luvas e logo que possível lavar bem as mãos com água e sabão ou utilizar o gel anti-séptico se disponível.
3. Utilizar testes e lancetas novos para cada pessoa pois a contaminação biológica pode conduzir a resultados incorrectos.
4. Seguir sempre os procedimentos operativos recomendados para cada técnica / operação.
5. Inutilizar o material utilizado no contentor apropriado:
    - Contentor de Biosegurança: para eliminar o material corto-perfurante (ex.: lancetas, lâminas);
    - Saco azul de lixo: para eliminar material contaminado com sangue, urina ou fezes;
    - Saco preto de lixo: para eliminar lixo comum (material não contaminado, ie, papeis, embalagens de cartão ou plástico).

4.2. Testes de Diagnóstico rápido – Considerações Gerais

Adicionalmente, teremos de lidar diariamente com TDRs que possuem regras próprias de manuseamento e cuidados gerais. Deste modo:

a) Conservação do material

- Os kits de testes de diagnóstico rápido (TDR) da malária devem ser conservados à temperatura indicada na embalagem, mantendo-o a uma temperatura constante.
- Manter as caixas fechadas e com o dessecante.
- Registar a data de abertura dos kits.
- Não congelar os componentes do kit.

b) Precauções

- Verificar o prazo de validade do kit e utilizar apenas se este estiver dentro do prazo.
- Não utilizar kits que estejam visivelmente danificados.
- Não reutilizar nenhum dos componentes dos kits (cassetes, pipetas, tiras…) pois a contaminação biológica pode conduzir a resultados incorrectos.
- Para a obtenção de resultados correctos têm que se seguir rigidamente estas instruções.
- O pessoal de laboratório deve usar luvas durante a colheita e o processamento das amostras biológicas. Troque as luvas sempre que se contaminarem ou considerar necessário.
- O material **corto-perfurante** (lancetas) é de uso único. Após a sua utilização, estes devem ser descartados em contentores de bio-segurança adequados. Não deitar para lixo comum.

c) Identificação

- **Amostras -** Todas as amostras recebidas devem ser imediatamente identificadas com a ID permanente do membro do agregado familiar seleccionado. Para o efeito, registar a ID Permanente completa nos contentores da urina e fezes com o marcador de ponta fina.
  NOTA: os contentores de fezes devem ser marcados pelo Trabalhador de Campo avançado no dia de entrega dos frascos aos participantes
- **Testes –** Todos os TDR´s usados devem ser identificados com a ID permanente do membro do agregado familiar. Assim, após abertura dos kits, deve de imediato escrever-se com o marcador de ponta fina a ID Permanente completa no kit ou teste, em local visível fora da zona reagente, de forma a evitar trocas.

5. Organização do Trabalho de Campo

O trabalho de campo em cada local de amostragem do **ramo comunidade**, será organizado do seguinte modo:

- **1º dia:** entrega de folhetos informativos/termos de consentimento e frascos para recolha de fezes aos Agregados familiares seleccionados (Trabalhador de Campo avançado e Motorista);
- **2º dia:** recolha dos dados (Equipa de campo) **3º dia:**;
- **3º dia:** Preenchimento do inquérito de efeitos secundários ás crianças de idade pré-escolar (2 a 5 anos) que foram medicadas na véspera

O trabalho inicia-se na segunda-feira com a visita do Trabalhador de campo avançado (entrega de folhetos e frascos de fezes), na terça-feira, quarta-feira e quinta-feira realizam-se as saídas de

campo da equipa de campo aos locais visitados pelo trabalhador de campo avançado na véspera e na sexta feira só o trabalhador de campo avançado sai para o campo para preencher os questionários de efeitos secundários

O trabalho de campo em cada local de amostragem do **ramo escola**, será organizado do seguinte modo:

- **1º dia:** Reunião com os Pais/responsáveis das crianças na escola e entrega de folhetos informativos/termos de consentimento e frascos para recolha de fezes (Coordenador, Trabalhador de Campo avançado e Motorista);
- **2º dia:** recolha dos dados (Equipa de campo)**.**

Periodicamente, serão organizadas reuniões com o todo o pessoal de forma a avaliar o andamento dos trabalhos, identificação de pontos fortes e fracos e planeamento futuro.

5.1. Circuitos de pessoas, amostras e registos

O esquema abaixo reproduzido, sistematiza a intervenção por bairro no **ramo comunidade**, identificando intervenientes, postos de trabalho, circuitos e tarefas globais:

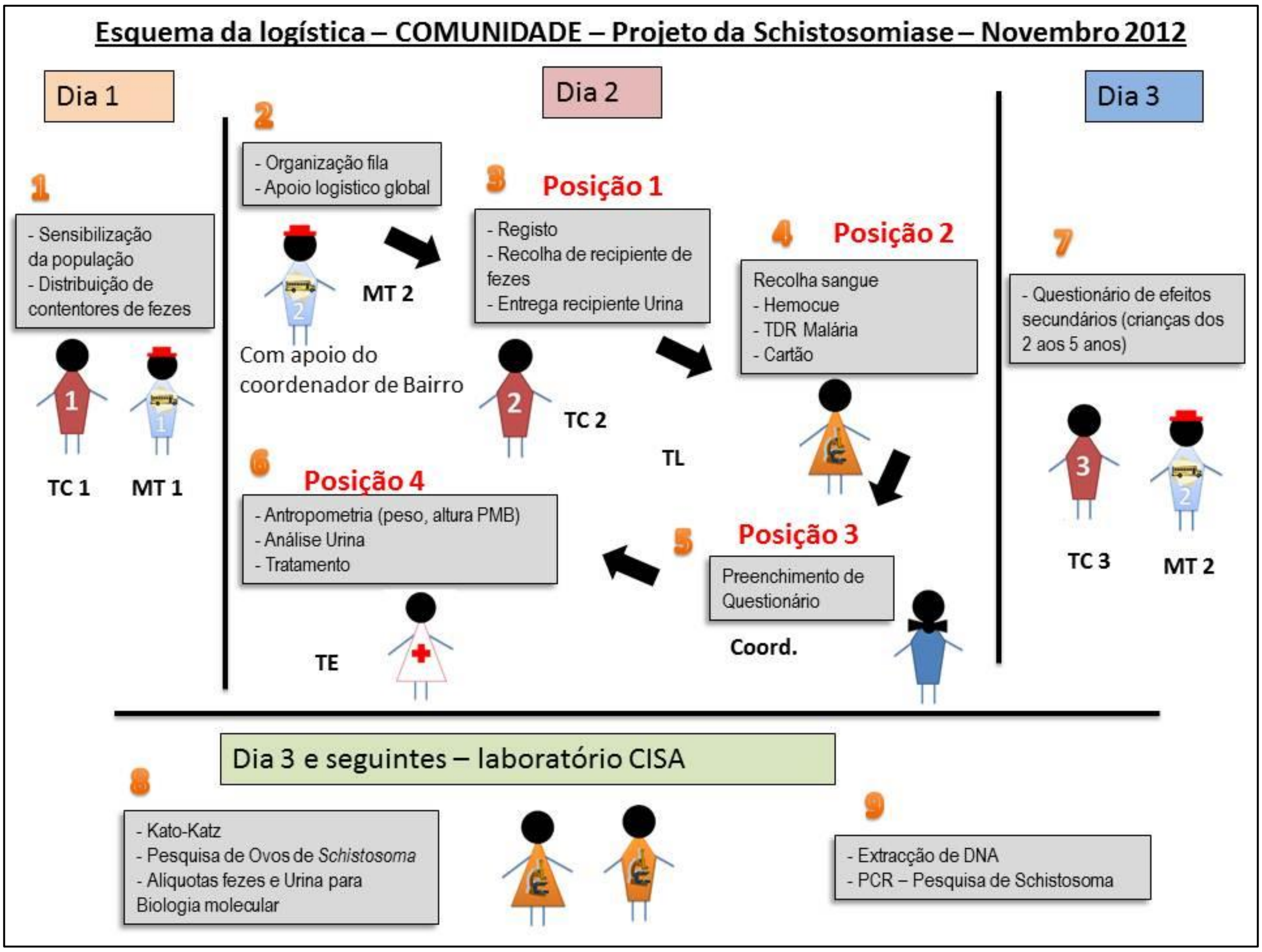

O circuito nos dias de recolha de dados, entre as diferentes posições, de pessoas, amostras e registos. Deste modo:

- **Crianças com acompanhante:** Com o apoio do Coordenador de Bairo, o Trabalhador de Campo e o Motorista deverão confirmar as presenças das pessoas a inquirir previamente contactadas e organizá-las numa fila frente à **posição 1**, onde será feito o seu registo, recolha de frascos de fezes e entrega de frascos para recolha de urina e do questionário (que deve acompanhar a criança ao longo de todo o circuito, até chegar à enfermeira que o recolhe). Posteriormente serão testadas na **posição 2** e após o término dos TDR e obtenção de resultados nesta mesa serão encaminhadas para a **posição 3**, onde realizarão o questionário, e finalmente serão

pesadas, medidas e medicadas na **posição 4**, recebendo os impressos de prova de tratamento e diagnóstico e eventuais impressos de encaminhamento para unidade de saúde.

- **Folhetos informativos/termos de consentimento:** Serão distribuídos aos seleccionados pelo **Trabalhador de Campo avançado** na visita prévia ao bairro, e deverão ser entregues ao Trabalhador de Campo na altura do registo na **posição 1**. Aí deverá ser confirmado que o inquirido percebeu o seu conteúdo e que foram assinados ou marcados com a impressão digital do responsável pela criança e posteriormente arquivados.
- **Frascos de fezes**: Serão distribuídos (devidamente identificados) aos inquiridos pelo **Trabalhador de Campo avançado** na visita prévia ao bairro, e deverão ser entregues ao Trabalhador de Campo na altura do registo na **posição 1,** com a quantidade pretendida de fezes. Aí deverão ser identificados e devidamente armazenados em arca.
- **Questionário:** entregue ao responsável da criança na **posição 1**, devendo acompanhar esta ao longo de todo o processo. Na **posição 2** servirá para registar os dados clínicos resultantes dos TDR da malária e da hemoglobina, na **posição 3** será preenchido pelo coordenador que entrevistará o responsável da criança e na **posição 4** será recolhido pelo Técnico de Enfermagem que consultará os dados clínicos já recolhidos para proceder ao tratamento e preencherá os dados de peso, altura e PBM em falta.
- **Frascos de urina:** Serão entregues aos participantes na **posição 1**, devendo ser devolvidos pelos próprios na **posição 4** com urina no seu interior para aplicação do teste de análise de urina.
- **Amostras de sangue em papel:** Serão colhidas na **posição 2** e posteriormente arquivadas para transporte para o **laboratório CISA**.
- **Impressos de tratamento e encaminhamento:** documentos que servirão como prova de tratamento e diagnóstico que deverão ser entregues na **posição 4** pelo Técnico de Enfermagem a todos os inquiridos, e documentos de encaminhamento para casos de anemia, malnutrição e outras patologias.

Para o grupo de crianças entre os 2 e os 5 anos (amostradas exclusivamente no ramo comunidade) a amostragem terá duas variantes. As primeiras 500 crianças amostradas serão medicadas para tratamento das parasitoses. No segundo grupo de 500 crianças amostradas, só serão tratadas as crianças com teses positivos para as parasitoses. Assim sendo uma equipa formada pela Técnica de enfermagem, pelo Trabalhador de Campo e pelo motorista, visitarão posteriormente o local de

amostragem para administrarem tratamento ás crianças positivas para as parasitoses. O resto do trabalho será idêntico. Todo o esquema encontra-se em baixo.

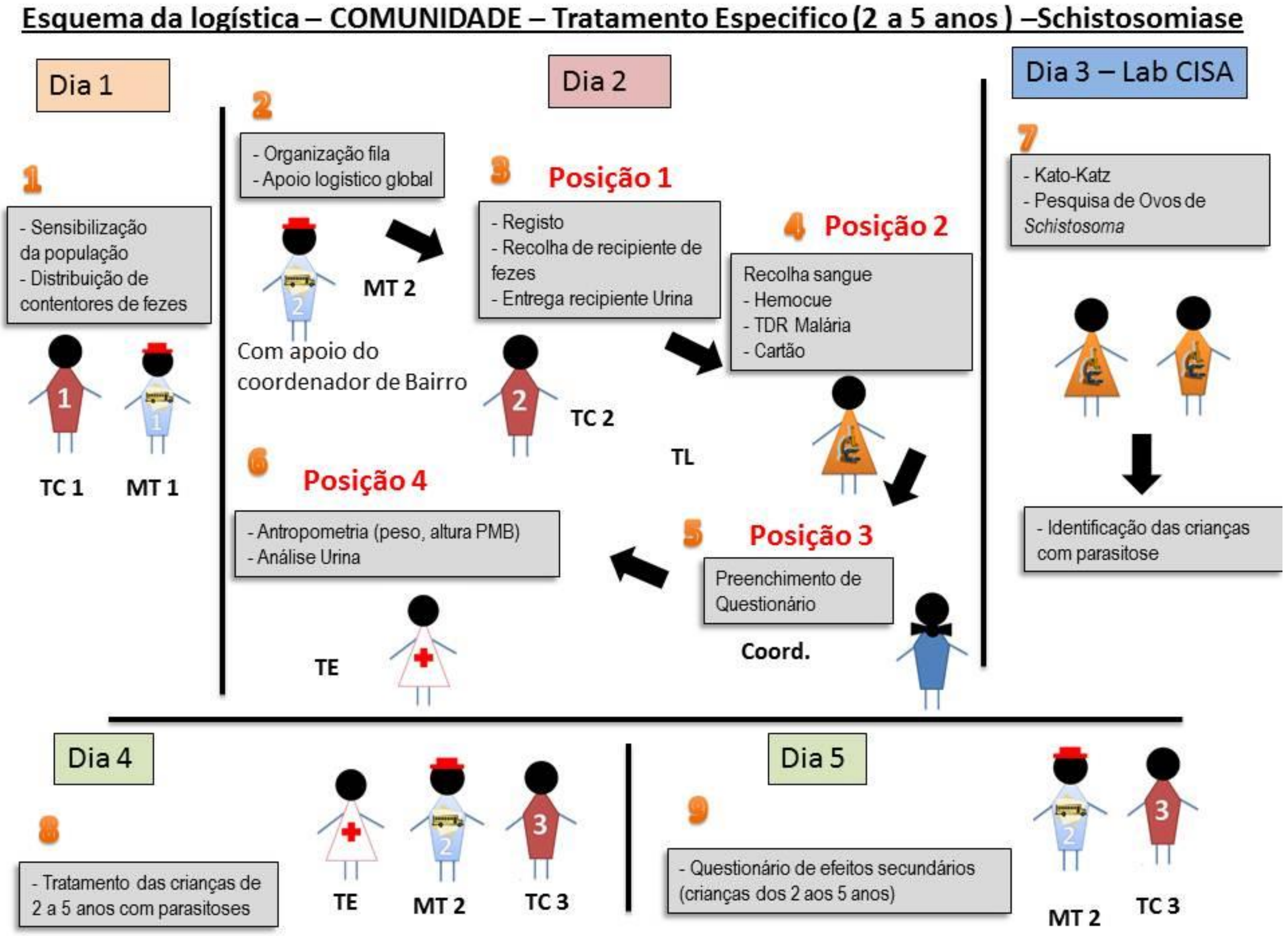

No que concerne ao ramo escola, o sistema de trabalho de campo é igual ao do ramo comunidade, no entanto o trabalho do dia anterior é distinto e não existe o questionário de efeitos secundários do dia posterior ás amostragens. O circuito geral está expresso na figura em baixo.

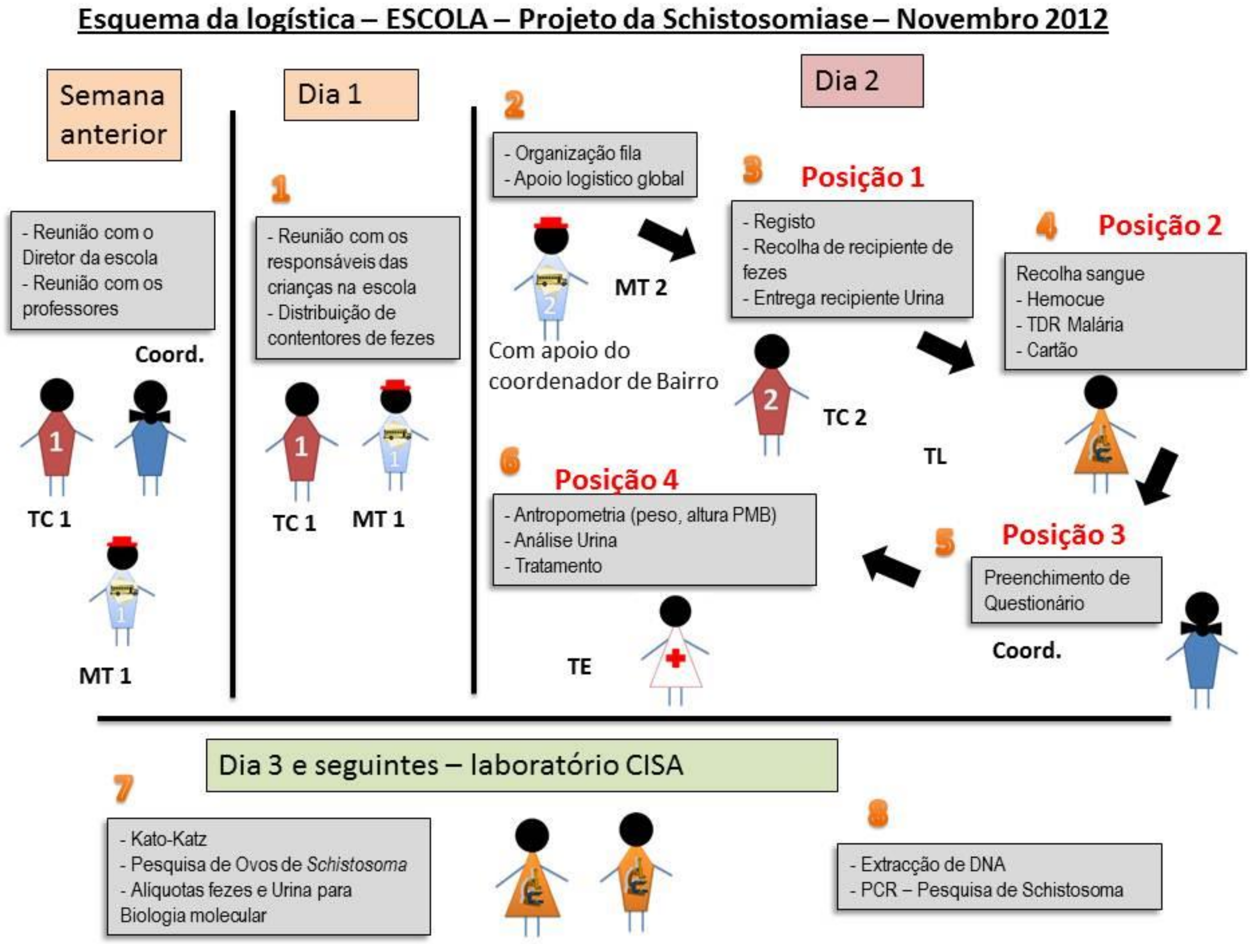

5.2. Cronograma-tipo Diário

O horário-modelo para o trabalho diário de implementação do inquérito no terreno, com a devida salvaguarda de alterações pontuais é o seguinte:

Hora	Actividade
	Conferência de material
8h00	Saída para o campo
	Montagem de mesas
	Conferência de documentação e amostras recolhidas
	Desmontagem de mesas
16h00	Regresso ao CISA
	Conferência de amostras entregues ao Laboratório CISA
	Conferência de documentação entregue e arquivo
	Preparação de material para dia seguinte

De notar que poderão haver situações em que será necessário ir para o campo antes das 8 da manhã ou regressar depois das 16h.

Será necessário efectuar refeições no local de trabalho nos bairros, sendo que esta é uma **responsabilidade de cada trabalhador** sem interferir no normal decurso do trabalho definido para esse dia.

Para facilitar o processo de preparação do material a levar para o campo, estarão disponíveis listas de controlo de material que devem usadas como instrumentos de apoio à preparação diária das actividades de campo (ver anexo).

6. Funções e Tarefas por Posição

Ao longo deste capítulo iremos abordar ao pormenor os materiais necessários, intervenientes e tarefas a desempenhar em cada um dos dias e nas posições de trabalho anteriormente definidas. A forma como as tarefas são apresentadas é sequencial e indicia uma ordem entre si.

a) Semana anterior á visita da equipa de Campo

RAMO COMUNIDADE

– Entrega de listas ao Coordenador de Bairro

Objectivo: Entrega da lista de agregados familiares seleccionados ao Coordenador de Bairro para que ele avise as pessoas respectivas da visita do projecto na semana seguinte (o dia será especificado) para entrega do folheto informativo e frasco para recolha de fezes. O Coordenador de Bairro poderá nesta altura determinar qual o local indicado para a equipa montar as operações no dia do inquérito.

- **Pessoal envolvido:** Motorista, Trabalhador de Campo avançado
- **Material necessário:** Listagem de agregados familiares seleccionados com os respectivos membros assinalados; carta pedido ao Coordenador de Bairro a solicitar aviso aos agregados familiares listados e pedido para o responsável de crianças entre 2 e 15 anos permaneçam em casa no dia combinado para receber o Trabalhador de Campo avançado.

RAMO ESCOLA

- Reunião com os Diretores de Escola e com os Professores

Objectivo: Explicação do projecto e organização da metodologia de reunião com os Pais e de recolha de dados.

- **Pessoal envolvido**: Coordenador do projecto**,** Motorista, Trabalhador de Campo avançado

b) 1º dia – Visita preliminar ao Bairro/Escola

RAMO COMUNIDADE

- **Objectivo:** Visita aos agregados familiares seleccionados, sendo fundamental para o sucesso das actividades a realizar nos dias de recolha de dados. Determinar o locar de montagem
- **Pessoal envolvido:** Trabalhador de Campo avançado e Motorista.
- **Material Necessário:**

  - Listagem de agregados familiares elegíveis com agregados familiares seleccionados e respectivos membros devidamente assinalados (estes devem ser visitados e recrutados) (ver **anexo**);

  - Folhetos informativos/Termos de consentimento

  - Frascos para recolha de fezes

  - Guia para colheita de amostras de fezes

  - Almofadas de carimbo

  · Marcadores permanentes

- **Tarefas a desenvolver:**

1. Identificação e aviso dos agregados familiares seleccionados para o inquérito e respectivos membros;
2. Entrega e explicação do estudo e dos termos de consentimento com recurso aos folhetos informativos – os membros seleccionados são livres de recusar, se tal acontecer pode explicar o estudo para tentar resolver algum mal-entendido mas se mesmo assim a pessoa recusar, o Trabalhador de Campo avançado deverá aceitar e passar para o agregado familiar seguinte.

   - nos que aceitarem participar a responsável pela criança deverá assinar ou marcar com impressão digital

   - o Trabalhador de Campo avançado deve também assinar e preencher os campos em branco (datas, local e **MUITO IMPORTANTE – o ID do agregado familiar**! Este número é que permite controlar o trabalho de campo!

- o Trabalhador de Campo avançado deve lembrar à responsável da criança para no dia seguinte comparecer no local combinado, e levar os Bilhetes de Identidade (BI) ou outro documento identificativo assim como cartões de saúde

3. Entrega de contentores de fezes e explicitação das regras de recolha de fezes com recurso ao Guia para colheita de fezes. Não esquecer de marcar os contentores com os IDs Perm de cada membro e explicar à responsável da criança qual frasco pertence a quem. Poderá ser necessário além do ID escrever um nome para facilitar a identificação dos frascos pela Mãe.
4. Para cada agregado familiar identificado o Trabalhador de Campo avançado deve marcar na Listagem de agregados familiares elegíveis qual o resultado da sua visita na coluna respectiva – se estava alguém em casa ou não, se entregou o Termo de consentimento e frasco para fezes; se deixou o aviso com o vizinho ou outro… Deve ainda assinalar com a sua caneta qual os membros que irão participar (com um "visto") e **informar do local do inquérito no dia seguinte**.
5. Ao fim do dia deve contar o total de agregados familiares que conseguiu visitar do total previsto para esse bairro

RAMO ESCOLA

Reunião com Pais e professor

- **Objectivo:** Visita á escola para apresentar o projecto aos Pais, e entregar frascos de recolha de fezes aos que autorizarem a participação dos filhos.
- **Pessoal envolvido:** Trabalhador de Campo avançado e Motorista.
- **Material Necessário:**
  - Listagem dos alunos
  - Folhetos informativos/Termos de consentimento
  - Frascos para recolha de fezes
  - Guia para colheita de amostras de fezes
  - Almofadas de carimbo
  - · Marcadores permanentes

- **Tarefas a desenvolver:**

1. Entrega e explicação do estudo e dos termos de consentimento com recurso aos folhetos informativos – os responsáveis da criança são livres de recusar, se tal acontecer pode explicar o estudo para tentar resolver algum mal-entendido mas se mesmo assim a pessoa recusar, o

Trabalhador de Campo avançado deverá aceitar a decisão. Nos que aceitarem participar a responsável pela criança deverá assinar ou marcar com impressão digital

- o Trabalhador de Campo avançado deve lembrar à responsável da criança para no dia seguinte comparecer no local combinado, e levar os Bilhetes de Identidade (BI) ou outro documento identificativo assim como cartões de saúde

2. Entrega de contentores de fezes e explicitação das regras de recolha de fezes com recurso ao Guia para colheita de fezes. Não esquecer de marcar os contentores com os IDs Perm de cada criança e explicar à responsável da criança qual frasco pertence a quem. Poderá ser necessário além do ID escrever um nome para facilitar a identificação dos frascos pela Mãe.
3. Para cada criança identificada o Trabalhador de Campo avançado deve marcar na Listagem de crianças elegíveis qual o resultado da sua visita na coluna respectiva Ao fim do dia deve contar o total de agregados familiares que conseguiu visitar do total previsto para essa escola.

c) 2º dia – Recolha de dados no Bairro/Escola

Neste dia, que corresponde a 3 dias da semana (terça-feira, quarta-feira e quinta-feira) serão efectuadas as amostragens e distribuídos os tratamentos.

No Grupo de crianças dos 2 aos 5 anos, só será entregue medicamentos nas primeiras quinhentas crianças. Nas restantes 500 crianças, as amostras de fezes e de urina serão analisadas no laboratório, e a equipa regressa dois dias depois ao local de amostragem para distribuir medicamentos **somente** ás crianças identificadas com parasitoses.

d) 3º dia – Preenchimento de inquérito de efeitos secundários em crianças dos 2 aos 5 anos (Só no ramo Comunidade)

- **Objectivo:** Realização de um questionário ás crianças entre os 2 e os 5 anos que no dia anterior foram medicados
- **Pessoal envolvido:** Trabalhador de Campo e Motorista.
- **Material Necessário:**
  - Listagem das crianças a entrevistar(ver **anexo**);
  - Questionários
- **Tarefas a desenvolver:**

Deslocar aos agregados familiares com crianças dos 2 aos 5 anos que na véspera foram estudados e que as crianças foram medicadas.

7. Trabalho efectuado em cada Posição

- **Posição #1 – registo e recolha de amostras**
- **Objectivo:** Organização das pessoas a inquirir e respectivo registo, recolha de amostras de fezes e termos de consentimento, entrega de questionário e preenchimento do cabeçalho e entrega de frascos para urina.
- **Pessoal envolvido:** 1 Trabalhador de campo
- **Material Necessário:**

- Contentores de amostras de fezes suplentes (x20)
- Contentores de urina (x70)
- Questionários (x70)
- Listagens de agregados familiares e membros seleccionados
- Almofadas de carimbo
- Marcadores Permanentes
- Folhetos informativos/Termos de consentimento

- **Tarefas a desenvolver:**

- Recolher termos de consentimento informado devidamente assinados ou com impressão digital se não estiverem preenchidos então explicar o estudo aos presentes e recolher os termos de consentimento informado depois de assinados.
- Conferência dos presentes com a Listagem de agregados familiares e membros elegíveis e seleccionados na coluna respectiva. Deve ter recebido esta listagem com as anotações do Trabalhador de Campo avançado (poderá ver se ele conseguiu no dia anterior avisar todos os agregados familiares ou quais os em falta).
-
- À medida que vai registando os participantes marcar com um "visto" na linha correspondente da listagem.
- Iniciar o preenchimento do questionário: ID do agregado familiar e crianças, bem como o seu nome completo, sexo e data de nascimento. Para esse preenchimento deve usar como auxílio quer as suas listagens de agregados familiares e membros seleccionados como os documentos de identificação que os participantes trouxerem consigo – deve pedir esses documentos à Mãe/Responsável pela criança.

- Entrega de questionário e informar que este deve acompanhar o participante até ao fim do inquérito.
- Rotular os frascos de colheita de amostras vazios de urina com o ID da criança, e entregar ao responsável pela criança e dar indicação para realizar colheita de amostra de urina das crianças para o frasco respectivo:

  Os frascos devem ser identificados com o número de identificação permanente completa do membro do agregado familiar seleccionado, com o marcador de ponta fina no frasco (não na tampa!). Entregar um frasco para cada uma das crianças seleccionadas. **Dar indicação para realizar colheita de amostra de urina:** "Urinar no copo e encher até ao meio, depois fechar bem o copo. Entregar todos os contentores na próxima mesa".
- Encaminhar os responsáveis e crianças para a próxima mesa (posição #2) informando que estas só serão atendidas após a urina ser recolhida.
- Receber as amostras de fezes, verificar o ID e armazenar na arca.

  Os frascos devem ter sido identificados com o número de ID permanente completo do membro do agregado familiar seleccionado, com o marcador de ponta fina pelo Trabalhador de Campo avançado (NO FRASCO, NÃO NA TAMPA!).

- Ao fim do dia o Trabalhador de Campo fixo deve contar quantas pessoas registou das que estavam previstas, informando o supervisor do ponto de situação.

Motorista

- Organização em parceria com o Coordenador de Bairro dos participantes presentes numa fila com grupos de mães/responsáveis e respectivas crianças. A prioridade é dada aos participantes que possuem os frascos com fezes, avisando os restantes que só são atendidos após as fezes estarem dentro dos frascos.

- **Posição #2 – Recolha de sangue periférico para análise**

Nota: para cada participante é muito importante que todos os TDR de Malária, couvetes do Hemocue e papeis de filtro tenham o mesmo ID que aquele que está escrito no questionário

- **Objectivo:** Colheita de sangue capilar e realização de TDR da Malária, quantificação de hemoglobina e recolha de sangue em cartão.
- **Pessoal envolvido:** 1 Técnico de Laboratório.
- **Material Necessário:**

- Cronómetro (x2), geleira, acumuladores de gelo
- Teste rápido malária: SD BIOLINE Malaria Antigen Rapid Test (x100)
- HemoCue (x2), cuvettes (x100)
- Lancetas, luvas, álcool a 70º, algodão
- Cartões com Papel de filtro Whatman, caixa plástica
- Contentor de biossegurança
- Ofertas para crianças (rebuçados)

Canetas, lápis, marcadores permanentes finos

- **Tarefas a desenvolver:**

1. Identificar todos o TDR de Malária a couvete do Hemocue e o cartão de papel de filro com a ID de cada participante.
2. Recolha de sangue capilar para:

   - TDR de Malária, com o kit SD BIOLINE Malaria Antigen Rapid Test

   - Hemoglobina, realizado pelo fotómetro HemoCue;

   - Papel de filtro **Whatman** (marcar o papel de filtro com o ID do participante)**;**
3. Armazenamento do material para enviar para o laboratório CISA, guardando papel de filtro (devidamente identificado).
4. Registar os resultados da Hemoglobina e TDR de malária no questionário,
5. Encaminhar as mães e crianças para a próxima mesa (posição #3).

- **Posição #3 – Realização do questionário**
- **Objectivo:** Recolha de dados respeitantes ás crianças e aos seus comportamentos e condições.
- **Pessoal envolvido:** Coordenador.
- **Material Necessário:**

  · Canetas
- **Tarefas a desenvolver:**

- Preencher o questionário com respostas obtidas da mãe.
- Encaminhar as mães e crianças para a próxima mesa (posição #4).

- **Posição #4 –Medição de peso, altura e PBM, teste de Urina e administração de terapêutica**

- **Objectivo:** Recolha de dados clínicos de mãe e crianças efectuar o teste da Urina com as fitas Combur 10 Test e administração de terapêutica a casos positivos.
- **Pessoal envolvido:** Técnico de Enfermagem.
- **Material Necessário:**
- Fitas de análise de urina Combur 10 Test, Roche (X100)
- Medicamentos:
    - Albendazol (x100)
    - Coartem (x10 para cada faixa)
    - Prazinquantel (x100)
    - - MTIs (mosquiteiros tratados com insecticida)
- Água (5 L), copos (x100)
- Luvas
- Ofertas para crianças (bolachas)
- Tabelas de tratamento e de %P/A
- Folha de diagnóstico e tratamento (x100)
- Impressos de encaminhamento (x100)
    - Anemia
    - Malnutrição
    - Malária e outras patologias
- Balança
- Estadiómetro
- Fita de medição de PMB
- Craveira para medição de altura infantil

  \- Agrafador

- **Tarefas a desenvolver:**
- Receber as amostras de urina e verificar se as IDs dos frascos são iguais às do questionário.
- Realização dos testes de fita Combur 10 Test, Roche às amostras de urina, conforme procedimento a seguir descrito. E registar os resultados no questionário

- Efectuar as medições de peso e altura de das cdianças e do PBM das crianças com menos de 5 anos, inclusive. Registar os dados nos respectivos espaços no questionário.
- Verificar os diagnósticos dos TDR (quadro 8) já registados no qustionário
- Com base nos valores medidos de peso, altura e PBMA e nos resultados dos TDR de Malária e Hb, preencher o impresso de prova de tratamento e diagnóstico para todos os membros inquiridos.
- Com base nesses valores, administrar a medicação de acordo com as seguintes recomendações:
  - · Coartem: tratamento padronizado de acordo com o peso [<14 kg (<2 anos), 6 comprimidos; 15 a 24kg (3-7 anos), 12 comprimidos; 25 a 34 kg (8 a 11 anos), 18 comprimidos; >35 kg (>= 12 anos), 24 comprimidos)] (ver anexo). Não administrar se TDR positivo mas tomou coartem na última semana.

Administrar a todas as crianças

Albendazol: dose única de 1 comprimido mastigável de 400 mg. Não administrar em mulheres grávidas ou a amamentar. Certificar que os participantes engolem os comprimidos.

Prazinquatel: distribuição de uma dose única de acordo com a tabela em anexo e de acordo com o peso do inquirido. Não administrar a crianças com menos de 4 anos e a mulheres a amamentar.

.

- Preencher os impressos de encaminhamento para casos de anemia, malnutrição e casos suspeitos de Malária ou outras patologias, para posterior informação do pessoal da unidade de saúde mais próxima. de acordo com as seguintes recomendações:
  - · Casos com anemia: Hb inferior a 9,9 g/dl;
  - · Malnutrição: P/A<70% (consultar tabelas anexas) ou/e PBM<13,5 cm em crianças com menos de 5 anos;
  - · Malária ou outras patologias: TDR positivo, sintomas de malária e tomou Coartem na última semana; ou TDR negativo e sintomas de malária.
- Arquivar todos os questionários recebidos.

8. Procedimentos Laboratoriais

Este capítulo destina-se em particular aos Técnico de Laboratório, e aos Técnico de Enfermagem. Pretende-se constituir uma fonte de consulta para o esclarecimento de qualquer dúvida ao longo dos procedimentos que decorrem no campo, e assegurar que todos os procedimentos de laboratório são efectuados com o rigor necessário de modo a obter resultados fiáveis.

8.1. Teste de análise de Urina – Técnico de Enfermagem na posição 4

Este processo inicia-se pela colheita e respectiva conservação das amostras de urina nos frascos fornecidos na posição 1. O Técnico de Enfermagem na posição 4 será responsável pela execução do teste de análise de urina pelas fitas Combur 10 Test .

Colheita e Conservação

1. Receber os frascos de urina dos participantes (Nota: devem ter no mínimo 10-15 ml de urina).
2. Se a urina se apresentar claramente conspurcada com fezes deve ser eliminada.
3. As análise devem ser realizadas até 1 hora após a colheita de urina
4. Registar os resultados na folha do questionário

a) Procedimento laboratorial para o fita Combur 10 Test - Pesquisa de microhematúria

Material necessário

- Tiras reagentes fita Combur 10 Test
- Contentor para colheita de amostra
- Cronómetro

Conservação do material

- As tiras de reagente Combur 10 Test devem ser guardadas à temperatura ambiente (15-30ºC).
- Manter a caixa fechada e com o dessecante.
- Registar a data de abertura do frasco. Após a abertura, as tiras podem ser usadas durante 6 meses, desde que a caixa permaneça sempre bem fechada.

Execução da técnica

1. Tirar uma tira, sem tocar na zona de teste, e fechar o frasco de imediato.
2. Marcar a tira com a ID permanente da pessoa seleccionada.
3. Mergulhar a extremidade da tira com o reagente na amostra de urina e remover a tira após 2 segundos. Ao remover a tira do contentor da amostra, passar com a tira no rebordo do contentor para eliminar excesso de urina. Segurar a fita enquanto espera que passem 1 a 2 minutos.
4. Efectuar a leitura do resultado após 1-2 minutos, comparando a cor da área de teste com a tabela de cores do rótulo do frasco. Não tocar com a tira no frasco para não o contaminar.

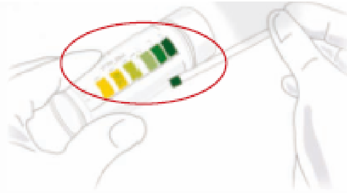

5. Registar todos os resultados do teste.

Resultados

Se a zona de teste não alterar de cor indica que o teste é negativo (NEG). A mudança de cor indica um resultado positivo e a intensidade da cor indica a quantidade de composto presente,.

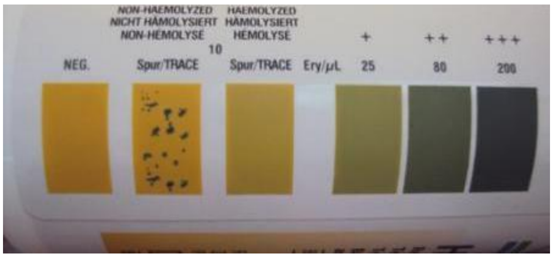

Registo dos Resultados: Registar o resultado do teste no campo correspondente do inquérito, no campo indicado:

b) Conservação de amostras de urina em parasitrap

As amostras de urina devem ser conservadas para se enviar para o laboratório, para posterior análise. Estas amostras devem ser conservadas, para impedir a eclosão dos miracídios (*Schistosoma*), o que torna os ovos indistinguíveis. Como proceder:

1. Adicionar imediatamente cerca de 0,1 ml de formalina não diluída (solução de formaldeído a 37%) por cada 10 ml de urina, com uma pipeta Pasteur (após ter feito o teste da Tira)
2. Conservar refrigerado (colocar na geleira com acumuladores de gelo) até serem levadas para o laboratório.
3. A técnica de filtração de urina será realizada no laboratório do Projecto CISA

8.2. Colheita de sangue capilar – Técnico de Laboratório da posição 2

Descrevem-se aqui os passos envolvidos na obtenção de amostra de sangue capilar por punção digital, aplicados para a colheita de amostras ás crianças.

- **Preparação geral**

1. Certifique-se que a superfície de trabalho (mesa), onde vai colocar os materiais de colheita e processamento das amostras, está perfeitamente limpa. Sempre que necessário, limpe a superfície com uma gaze embebida em álcool.
2. Disponha na superfície de trabalho todo o material necessário para a colheita e processamento das amostras, antes de iniciar o procedimento de colheita de amostra.
3. Marque o material com o numero de ID respectivo (Couvet do Hemocue, Teste Malária, e cartão de papel de filtro).
4. . **Use sempre luvas** e observe as medidas de segurança quando efectua a colheita de material biológico. Troque as luvas sempre que se contaminarem e lave sempre as mãos quando necessário.
5. Quando a colheita for realizada descreva aos pais/encarregados exactamente o que será feito durante a colheita da amostra de sangue e como eles podem ajudar, por exemplo manter a criança no seu colo e pegar na mão da criança durante a colheita da amostra.
   A criança pode estar com medo ou ansiosa sobre o que vai acontecer, por isso é importante usar uma forma de a acalmar à medida que se começa a efectuar a colheita de amostra de sangue. Lembre-se que a comunicação não verbal é importante, por exemplo manter o contacto no olhar com a criança quando estiver a preparar para tirar a amostra.

Material necessário para a punção capilar

- **Lancetas BD Microtainer:** a lanceta é um dispositivo de incisão automático descartável usado para punção no dedo. O dispositivo possui uma agulha de silicone revestida para mais facilmente se adaptar à pele minimizando a dor. Quando a alavanca é pressionada, uma agulha sobressai rapidamente de dentro do dispositivo. Possui ainda um mecanismo de segurança que recolhe a lanceta após o uso o que impede a picada acidental do técnico.
- **Luvas de látex** - usadas para reduzir o risco de doenças de origem
  sanguínea. As luvas devem ser colocadas pelo Técnico de Laboratório
- **Toalhete com álcool descartável**- limpar a pele antes da picada do dedo com o toalhete que vem no Kit SD Malária Ag P.f/P.v.

- **Contentor de Biosegurança** – para eliminar o material corto-prefurante.
- **Algodão**- para limpar os dedos em caso de sujidade antes da colheita

Identificação: O membro do agregado familiar deve ser identificado com o número de ID permanente antes de se realizar a colheita da amostra de sangue e de seguida identificar todo o material.

Punção capilar

a) Selecção do local da punção - Punção digital

- A colheita torna-se mais fácil se estiver sentado ao lado da pessoa a quem se vai efectuar a colheita de sangue. Por exemplo, se pretender efectuar a colheita de amostra na mão esquerda, deve posicionar-se no lado direito do indivíduo.
- **Usar o dedo médio ou anelar** para a colheita de sangue.

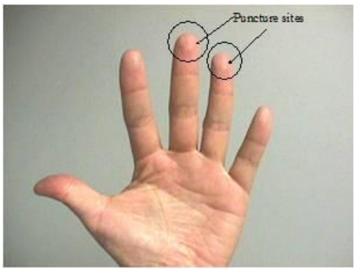

c) Desinfecção da pele

- Desinfectar **cuidadosamente a pele com o toalhete com álcool descartável**. Se a pele estiver muito suja, use um segundo algodão.
- Deixar o álcool secar antes da punção pois o álcool residual causa hemólise e pode interferir com os resultados. Não soprar para fazer secar o álcool porque este procedimento pode contaminar o local desinfectado com bactérias.

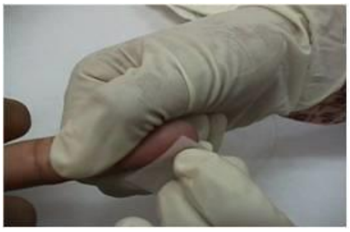

d) Punção

- **Certificar que o dedo a puncionar esteja situado abaixo do nível do coração** para aumentar o fluxo de sangue para o dedo. Com o polegar, massajar levemente o dedo do nó para a ponta, **mantendo uma pressão suave.** Esta acção estimula o fluxo de sangue para o ponto de colheita da amostra
- Pode ser útil se os pais/encarregados ajudarem, segurando as mãos da criança.

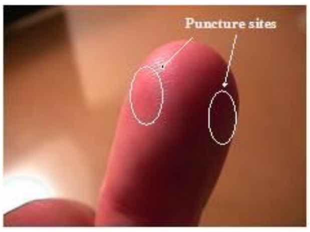

- Para **melhor fluxo de sangue e causar menos dor, posicionar a lanceta ligeiramente afastada do centro da polpa do dedo, perpendicularmente à superfície da pele, e** não no centro da ponta **do dedo. Evitar a extremida**de da ponta do dedo ou os lados para além da zona da palma do dedo.

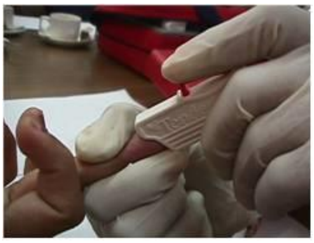

- Usar a lanceta para **perfurar a pele, numa acção rápida** produzindo uma micro incisão na pele. Imediatamente a agulha se retrai para o interior do dispositivo. Depois da picada da pele, vire o dedo ligeiramente para evitar que o sangue escorra entre os dedos.

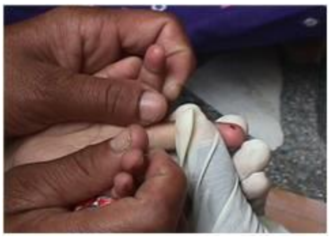

- Depois de usar, as lancetas devem ser descartadas no contentor de segurança biológica e o material contaminado não cortante deve ser colocado no saco de lixo biológico.

e) Colheita da amostra de sangue

- Quando o sangue aparecer, fazer em primeiro lugar a gota para papel de filtro.

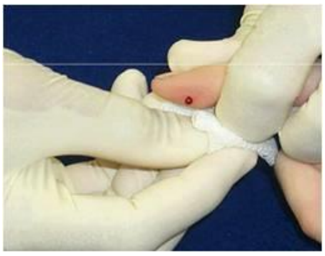

- O fluxo sanguíneo aumenta se colocar o local da punção para baixo e aplicar pressão suave no tecido circundante (ou próximo ao local da punção quando o sangue é obtido de um dedo). Periodicamente, aliviar a pressão no dedo para permitir a passagem do fluxo sanguíneo
- Efectuar a colheita da segunda gota para o **Kit SD Malária Ag P.f/P.v.**, depois colheita para **teste de hemoglobina**, no fim nova gota para o *papel de filtro*. Notar que tem de ser rápido nos procedimentos, caso contrário o sangue irá coagular no dedo.
- Se o sangue parar de fluir antes que uma quantidade suficiente tenha sido colhida, o procedimento da picada na pele pode ser repetido com o consentimento do indivíduo ou pais/encarregados num dedo diferente. Não reutilizar nenhum dos materiais usados na primeira picada.
- Após a colheita, manter o local da picada elevado e com o algodão, aplicar uma ligeira pressão até deixar de sangrar, cerca de 1-2 minutos.

8.3. Procedimento Laboratorial do Kit SD Malária Ag P.f/P.v.–

Técnico de Laboratório e Trabalhador de Campo na posição 2

Material necessário

- no Kit SD Malária Ag P.f/P.v., que inclui:
    - Cassete do teste Paracheck Pf ®
    - Aplicador da amostra (semelhante a uma ansa) de 5 µl
    - Lanceta
    - Reagente tampão
- Cronómetro

Conservação do material

- O Kit **Paracheck Pf** ® pode ser guardado à temperatura de 4-45ºC.
- O kit deve se colocado à temperatura ambiente antes de ser usado para o teste. Caso o kit tenha sido armazenado a uma temperatura de 2 – 8ºC, esperar que o material recupere a temperatura ambiente durante cerca de 30 minutos.

Execução da técnica Paracheck $_{Pf}$ ®

1. Abrir a embalagem do teste e retirar a tira e o aplicador, sem tocar na zona de teste. Verificar a cor do exsicador presente na embalagem: deve ser azul, se estiver incolor ou rosado deve ser usada uma nova tira de teste;
2. Identificar a cassete de teste com a identificação permanente (ID) do membro do agregado familiar (AF).
3. Rodar a tampa do frasco de tampão fornecida com o kit no sentido dos ponteiros do relógio para furar a abertura do frasco conta-gotas;
4. Tocar com o aplicador da amostra sobre a 2ª gota de sangue que se forma sobre a picada no dedo (a 1ª não é utilizada). Assim que a ansa do aplicador se encher de sangue, retirar o aplicador e colocar a amostra obtida no **poço A** da cassete do teste:

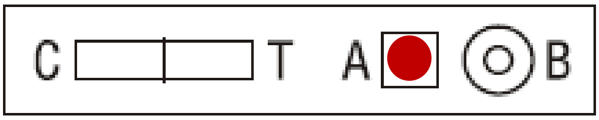

A amostra deve ser colocada no poço do teste imediatamente após a colheita para impedir a coagulação do sangue. **Nota** - Assegurar que o sangue do aplicador foi todo depositado no poço!

5. Com o frasco de reagente tampão na vertical, dispensar 6 gotas no **poço B**; Este procedimento deve realizado com a **ajuda do trabalhador de campo** (**TC**): o Trabalhador de Campo deve colocar as 6 gotas de reagente imediatamente após o Técnico de Laboratório ter colocado o

sangue no dispositivo de teste e iniciar a contagem do tempo no cronómetro. Assim, o Técnico de Laboratório poderá continuar a recolha de sangue para os outros testes.

6. Efectuar a leitura do resultado após 15 minutos.
7. Inutilize o material utilizado no contentor apropriado – lanceta colocar no contentor de segurança biológica e material contaminado com sangue colocar no lixo. Utilize novo teste e lanceta para as pessoas seguintes.

Interpretação dos resultados: O resultado deve ser lido 15 minutos após se ter colocado a amostra e reagente na cassete do teste. Qualquer banda que apareça após este período deve ser ignorada.

Resultado Positivo para malária por *P. falciparum*

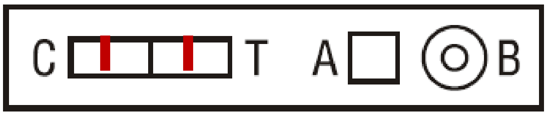

C- linha de controlo interno do teste
T – linha do teste

O teste é positivo se aparecerem duas linhas na membrana da cassete. Qualquer banda colorida rosa na região do teste indicará um resultado positivo. O resultado do teste é positivo mesmo quando a linha de teste tem uma aparência mais clara ou mais escura do que a linha de controlo.

Resultado Negativo para malária por *P. falciparum*

C- linha de controlo interno do teste

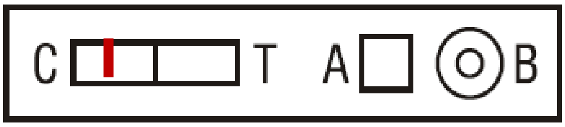

T – linha do teste

O teste é negativo se apenas a linha de controlo for visível. Para ter a certeza de que as amostras com resultados positivos baixos tiveram tempo suficiente de processamento, os resultados negativos devem apenas ser registados 15 minutos após se ter colocado a tira no tubo contendo o reagente.

Resultado Inválido

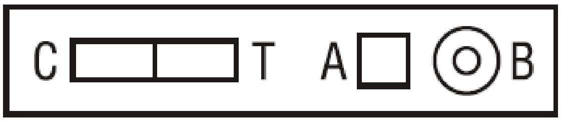

O teste é inválido se a linha de controlo não for visível. Se isto acontecer, deve-se repetir o teste.

Informe a pessoa testada do resultado do teste, se positivo terá de ser tratada pela enfermeira que faz parte da equipa

Registo dos Resultados: Registar o resultado do teste no campo 8 do inquérito, no campo indicado: (marque com uma cruz (X) a opção correcta)

8. Resultados Laboratoriais no Campo (Testes rápidos) – A preencher na posição #2

	Teste		Resultado	T Lab
Mãe IDPerm □□□	Urina	Hematúria macroscópica	□ Sim □ Não	
		Microhematúria - Hemastix	□ (-) □ (vestígios sg não hemolisado) □ (vest. sg hemolisado) □ (+) □ (++) □ (+++)	
		CCA	□ Positivo □ Negativo	
		Urina guardada para filtração?	□ Sim □ Não	
	Sangue	Hemoglobina (Hb - g/dl)	______ g/dl	
		Paracheck	□ Positivo □ Negativo	

8.4. HemoCue ® - Determinação da hemoglobina
Técnico de Laboratório da posição 2

a) Descrição do equipamento HemoCue® - Componentes

1. Analisador HemoCue Hb 201+.

2. Transformador de corrente.

3. 4 Pilhas de tipo AA ou R6, 1.5 V.

4. Embalagem de microcuvettes HemoCue Hb 201

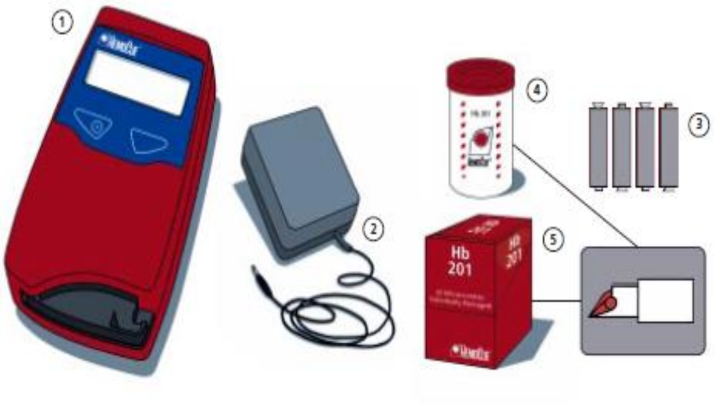

b) Armazenamento e conservação

Microcuvetes HemoCue Hb 201

As cuvetes são sensíveis a humidade. Deve-se observar os seguintes cuidados para um adequado manuseamento e armazenamento das cuvetes:

1. Usar as microcuvetes HemoCue Hb 201 até à data de validade impressa em cada embalagem. As cuvetes mantêm-se estáveis durante dois anos a partir da data de produção.

2. Após a abertura da embalagem, as cuvetes permanecem estáveis por 3 meses. Assim que se abre uma nova embalagem, a data deve ser registada no rótulo.
3. Retirar da caixa apenas as cuvetes de análises necessárias para o teste.
4. Manter sempre a embalagem devidamente fechada.
5. As cuvetes devem ser armazenadas à temperatura ambiente (15 – 30 °C). Não refrigerar e evitar a sua exposição ao aquecimento ou a forte luz do sol.

Analisador HemoCue Hb 201+

1. O analisador pode ser armazenado a 0 – 50°.
2. A temperatura de trabalho é de 18 a 30°C. Permitir que o analisador atinja a temperatura ambiente antes de o usar.

c) **Amostras:** No presente estudo, a amostra a testar é a obtida por punção capilar.

d) Medição da hemoglobina em sangue capilar

Material necessário

- Analisador HemoCue Hb 201+
- Microcuvettes HemoCue Hb 201
- Lancetas
- Toalhetes com álcool descartáveis
- Papel higiénico

Processamento de amostra de sangue capilar

1. Ligar o equipamento
   a. Ao ligar, o porta-cuvete deve estar na sua posição de carga.
   b. O ecrã mostra três traços a piscar e o símbolo HemoCue.

2

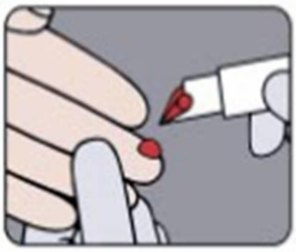

2. Quando a gota de sangue obtida por punção capilar for suficientemente grande, tocar com a cuvete na amostra e encher num processo contínuo. NÃO tornar a encher! (neste caso será a 3ª gota – a 1ª é para o papel de filtro, a 2ª é para o Kit SD Malária Ag P.f./P.v. a 3ª para o Hemocue e a ultima de novo para o papel de filtro). A amostra de sangue com cerca de 10 µl é introduzida na cavidade da cuvete por acção capilar.

3

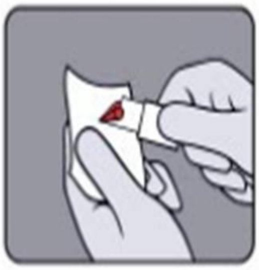

3. Limpar o sangue em excesso fora da ponta da cuvete, com papel higiénico. Assegurar que não saiu nenhum sangue da cuvete durante este procedimento. **Nota:** A cuvete foi concebida para conter a quantidade exacta de sangue necessária para o teste. É importante assegurar toda a ponta da lâmina de análise (ambos os círculos e a ponta) coberta com reagentes, esteja preenchida com sangue capilar.

4

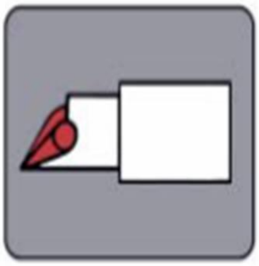

4. Verificar se há bolhas de ar na cuvete cheia. Se houver, encher outra cuvete. Bolhas à volta da ponta podem ser ignoradas.

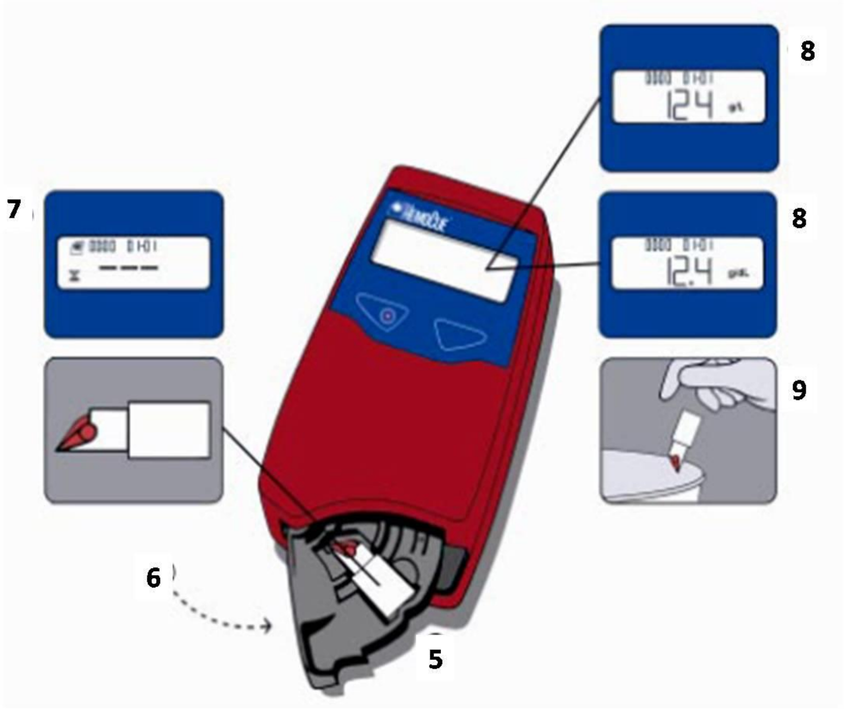

5. Colocar a cuvete cheia no porta-cuvetes. Isto deve ser realizado até 10 minutos depois de encher a cuvete!

6. Empurrar o porta-cuvete para a sua posição de medição, até fazer um estalido.

7. Durante a medição aparecerá ”"” no ecrã. Aguarde enquanto o instrumento mede a absorção da luz e apresenta os resultados no ecrã.

8. Ao fim de 15 – 60 segundos o valor da hemoglobina da prova será exibido no ecrã. O resultado permanecerá no ecrã enquanto o porta-cuvetes estiver na posição de medição. Se estiver a trabalhar a pilhas, o analisador desliga-se automaticamente depois de uns 5 minutos de inactividade e neste caso voltar a ligar o equipamento.

9. Registar o resultado da hemoglobina no questionário, retirar a cuvete de reacção e colocá-la no contentor de resíduos biológicos.

Nota: Todos os resultados são guardados no analisador, podendo ser vistos no ecrã usando a função de "scroll" (para mais informações consultar o manual do equipamento).

Registo dos Resultados: Registar o resultado do teste no inquérito, no campo indicado:

Controlo de Qualidade

O analisador HemoCue Hb 201+ dispõe de um "AUTO-TESTE" electrónico. Cada vez que se liga o analisador, ele verifica automaticamente a realização da sua unidade optrónica. Se o analisador se mantiver ligado, o teste é realizado de duas em duas horas.

Diariamente e sempre que se abre uma **nova embalagem** de cuvetes, o sistema HemoCue deve ser verificado com controlos Hemotrol, alternando entre o nível baixo e nível normal. Permitir que os controlos atinjam a temperatura ambiente antes de se executar o teste de controlo de qualidade (CQ).

1. Com o equipamento ligado (ao ligar, o porta-cuvete deve estar na sua posição de carga e o ecrã mostra três traços a piscar e o símbolo HemoCue), premir ambas as teclas simultaneamente.
2. O ecrã exibe um símbolo QC a piscar.
3. Seleccionar o teste QC premindo a tecla da esquerda.
4. O analisador retorna automaticamente para a sua posição de medição e o símbolo QC aparece no ecrã.
5. Encher a cuvete com solução de controlo recomendada pela HemoCue e executar as medições como se de uma amostra se tratasse, de acordo com os passos 12 – 16 referidos no capítulo anterior.
6. Uma vez efectuada a medição, o analisador volta automaticamente à sua posição de medição, desaparecendo do ecrã o símbolo QC. Pode começar o teste ao doente.

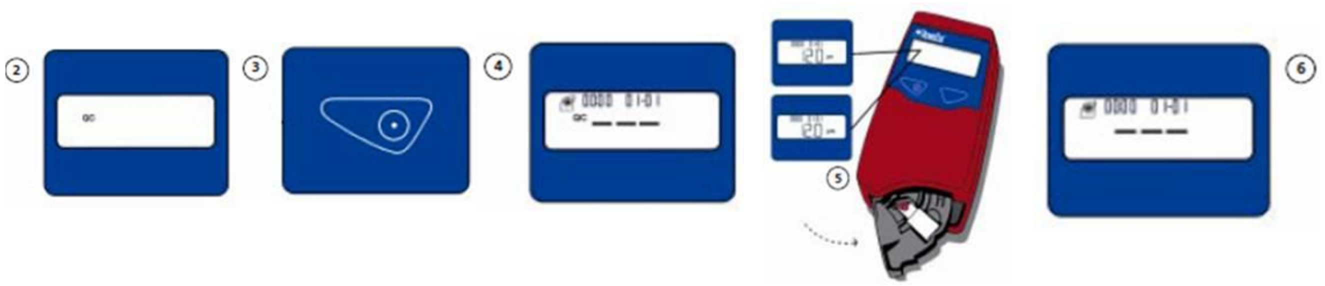

Nota: Se for necessário executar mais testes de QC, proceder da mesma maneira seguindo os passos 1 – 6.

Para desactivar o teste QC:

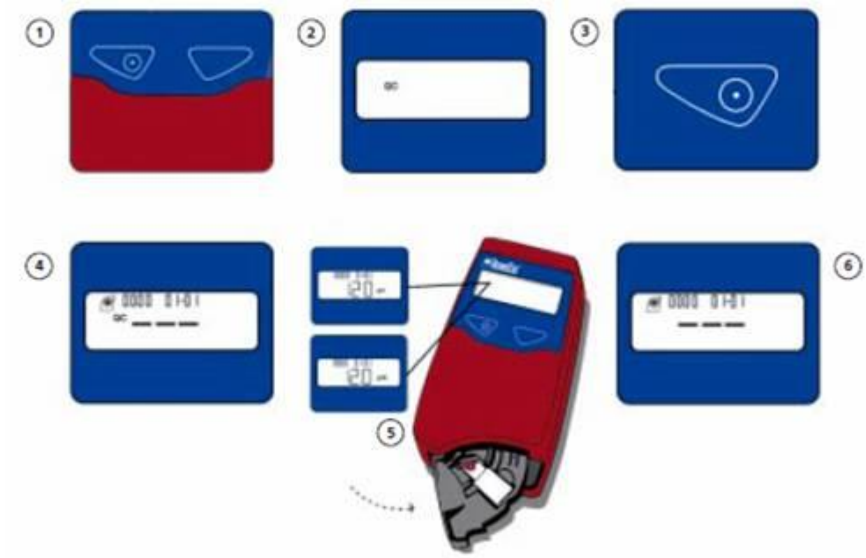

1. Premir ambas as teclas simultaneamente.
2. O ecrã exibe um símbolo QC a piscar.
3. Pressionar a tecla da direita até aparecer no ecrã outra actividade de configuração (4). Segurar a tecla em baixo durante uns 5 segundos. O analisador retorna automaticamente à sua posição de medição e o símbolo QC não se vê mais.

Manutenção do equipamento

Limpeza exterior do equipamento: Realizar esta limpeza sempre que for necessário, limpando o equipamento com uma gaze embebida em álcool.

Limpeza do suporte das cuvetes: Realizar este procedimento **diariamente**, no final do dia de trabalho.

1. Verificar se o analisador está desligado.
2. Puxar o suporte das cuvetes (porta-cuvete) para a sua posição de carga. Usar um objecto afilado para baixar cuidadosamente o fecho situado no canto superior direito do porta-cuvete.

3. Mantendo o fecho *despressionado*, puxar cuidadosamente o porta-cuvete o mais possível para o lado esquerdo. Puxar cuidadosamente o porta-cuvete para o lado indicado pelo manípulo.
4. Limpar o porta-cuvete com álcool. NUNCA colocar algodão humedecido com água ou com álcool dentro do porta-cuvete. O porta cuvetes deve estar completamente seco antes de ser reinserido no fotómetro.

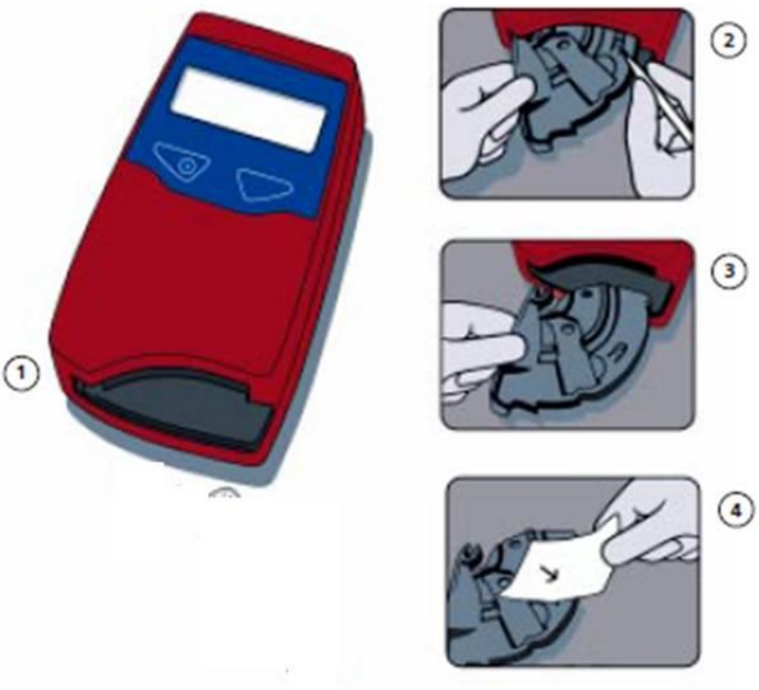

4.5. Conservação de amostras de fezes no frasco Parasitrap® -

Uma sub-amostra das amostras de fezes devem ser conservadas e enviadas para o laboratório para posterior realização da técnica de concentração

Material necessário do Kit Parasitrap®

· Tubo 1 de processamento da amostra
· Espátula
· Zaragatoa para aplicação da amostra

Procedimento

1. A amostra de fezes a ser conservada, deve ser colocada no Tubo 1 de processamento da amostra do Kit Parasitrap®, com o auxílio do êmbolo anexo à tampa de rosca. O tubo é bem fechado e agitado uma ou duas vezes (ver imagem).

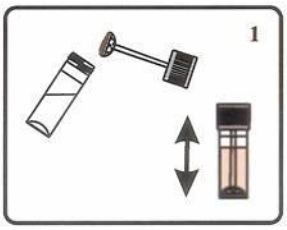

2. Conservar refrigerado até à entrega no laboratório (colocar na geleira com acumuladores de gelo).

5. Utilização das listagens de agregados familiares e membros seleccionados

As listagens de agregados familiares e membros seleccionados serão utilizadas pelo Trabalhador de Campo avançado (no dia anterior ao dia do trabalho de campo) e pelo Trabalhador de Campo no dia do trabalho de campo.

Apresenta-se uma breve explicação representativa de vários casos que podem acontecer, usando como exemplo o bairro Kinguxi. Consultando a listagem de agregados familiares elegíveis do bairro Kinguxi veríamos que tem 198 agregados familiares elegíveis (do total de 337 agregados familiares registados no SVD), tendo sido decidido que destes 198 apenas 36 agregados familiares vão ser inquiridos.

ID mebro	Nome	sex	Idade	Relação c/ chefe	entrega TdC/ frasco fezes	inquerito 1	inquerito 2
"KGX-00-001-001"	"PIEDADE JOAO GOVEIA"	"f"	52,77	"Chefe de agregado"			
"KGX-00-001-002"	"LUCRECIA NUNES JOAO"	"f"	32,57	"Filho/filha"			
"KGX-00-001-003"	"MARIA NUNES SALVADOR"	"f"	21,26	"Filho/filha"			
"KGX-00-001-004"	"JOAO NUNES SALVADOR"	"m"	16,81	"Filho/filha"			
"KGX-00-001-005"	"ANTONIO JOAO RUFINO"	"m"	14,03	"Neto/Neta"			
"KGX-00-001-006"	"PIEDADE JOAO RUFINO"	"m"	11,84	"Neto/Neta"			
"KGX-00-001-007"	"JOAQUIM JOAO RUFINO"	"m"	8,93	"Neto/Neta"			
"KGX-00-001-008"	"PAULO JOAO RUFINO"	"m"	5,86	"Neto/Neta"			
"KGX-00-001-009"	"ISABEL JOAO RUFINO"	"f"	3,09	"Neto/Neta"			
"KGX-00-001-010"	"AUGUSTINHO SALVADOR MIVO"	"m"	2,56	"Neto/Neta"			
"KGX-00-005-001"	"PEDRO GABRIEL MANUEL"	"m"	42,32	"Chefe de agregado"			
"KGX-00-005-002"	"NATALIA ANGELO"	"f"	20,55	"Esposa do chefe"			
"KGX-00-005-003"	"ANTONIO FELECIANO GABRIEL"	"m"	9,46	"Filho/filha"			
"KGX-00-005-004"	"JUDITH MADALENA ANGELO"	"f"	5,85	"Filho/filha"			
"KGX-00-005-005"	"ADAO DOMINGOS ANGELO"	"m"	4,09	"Filho/filha"			
"KGX-00-005-006"	"CHANA ANGELO CHIETA"	"f"	0,56	"Filho/filha"			
"KGX-00-006-001"	"ADAO DOMINGOS DIAINDO"	"m"	60,77	"Chefe de agregado"			
"KGX-00-006-002"	"JUDITH MADALENA ANTONIO"	"f"	39,3	"Esposa do chefe"			
"KGX-00-006-003"	"GOMES LUCIANO DOMINGOS"	"m"	15,77	"Enteado/Sobrinho"			
"KGX-00-006-004"	"ANTONIA DOMINGOS LUCIANO"	"f"	13,43	"Enteado/Sobrinho"			
"KGX-00-007-001"	"MARIA JOAQUIM GANCALO"	"f"	36,56	"Chefe de agregado"			
"KGX-00-007-002"	"MARIZA DEMBO ANTONIO"	"f"	16,01	"Filho/filha"			
"KGX-00-007-003"	"CATERINA SEBASTIAO SIMAO"	"f"	12,41	"Filho/filha"			
"KGX-00-007-004"	"MANILSA MANUEL VAN DUNEM"	"f"	5,9	"Filho/filha"			
"KGX-00-008-001"	"MADALENA JOAQUIM GONCALO"	"f"	24,3	"Chefe de agregado"			
"KGX-00-008-002"	"DIOGO JOAQUIM GONCALO"	"m"	3,31	"Filho/filha"			
"KGX-00-009-001"	"LUIS SANTOS DIOGO"	"m"	26,09	"Chefe de agregado"			
"KGX-00-009-002"	"ISABEL DAMIAO BOA"	"f"	26,77	"Esposa do chefe"			
"KGX-00-009-003"	"SANTOS SEBASTIAO GIOGO"	"m"	6,77	"Filho/filha"			
"KGX-00-011-001"	"LOPES DAMIAO PACATO"	"m"	36,49	"Chefe de agregado"			
"KGX-00-011-002"	"PAULINA AUGUSTO ADAO"	"f"	29,87	"Esposa do chefe"			
"KGX-00-011-003"	"AUGUSTO LOPES DAMIAO"	"m"	11,82	"Filho/filha"			
"KGX-00-011-004"	"DAMIAO LOPES PACATO"	"m"	9,3	"Filho/filha"			
"KGX-00-011-005"	"FELECIANA LOPES DAMIAO"	"f"	6,62	"Filho/filha"			
"KGX-00-011-006"	"JULHO LOPES DAMIAO"	"m"	2,9	"Filho/filha"			
"KGX-00-013-001"	"JOAO CASSANGE NGUNZA"	"m"	34,11	"Chefe de agregado"			
"KGX-00-013-002"	"JOANA MANUEL"	"f"	29,77	"Esposa do chefe"			

Caxito.BBC / Caxito.CBG / **Caxito.KGX** / Caxito.KJD / Caxito.KSM / Caxito.KTG / Caxito.KXQ / Caxito.PRN / Caxito.STN / Caxito.SSC / Mabubas.BE1 / Mabubas.CPD

Nas listagens fornecidas para cada bairro estão todos os agregados familiares elegíveis em cada bairro (ie, que têm pelo menos uma uma criança entre 1 e 15 anos) assim como todos os seus residentes. (neste exemplo os agregados familiares 001, 005, 006, 007, 008, 009, 011 e 013 são elegíveis mas não os agregados familiares 002, 003 e 004, que não aparecem nesta lista)

Estão ainda assinalados os agregados familiares que foram seleccionados dentro dos elegíveis (com um fundo cinza). (Neste caso, o agregado familiar 001, e 011 foram seleccionados para participarem enquanto os agregados familiares 005, 006, 007, 008 e 009 não o foram, embora também tenham membros elegíveis)

No caso do agregado familiar 011 há uma mulher/mãe e respectivas crianças (2 de cada faixa etária) que serão convidadas a participar.

No dia da visita preliminar o Trabalhador de Campo avançado deve procurar os agregados familiares seleccionados e recrutá-los para participarem no estudo (agregados familiares enquadrados por

uma linha mais grossa e com membros sombreados a cinzento na listagem). Deve assinalar na sua coluna “entrega de Termo de consentimento/frascos para fezes” o resultado da sua busca: se os encontrou e escolheu os membros participantes (a quem entregou frasco para recolha de fezes) deve assinalar essa linha com um “visto” (√); assim como outras situações – se não encontrou com um “X”, se avisou o vizinho da tentativa de contacto a quem deve pedir para mulher e crianças do agregado familiar seleccionado (não do vizinho!) se dirijam ao local do inquérito no dia seguinte. Os agregados familiares devem ser assinalados com um “visto” (“√”) ou “X” conforme o caso de terem sido encontrados e recrutados ou não.

Ao fim do dia deve passar esta tabela ao supervisor para informação ser partilhada com o Trabalhador de Campo fixo que irão usar essa listagem no dia seguinte.

Nota: **TODAS AS INFORMAÇÕES NESTAS LISTAGENS E RESULTADOS OBTIDOS NESTE INQUÉRITO SÃO CONFIDENCIAIS, ie, não podem ser utilizadas pelos trabalhadores deste estudo.**

6. Preenchimento do Questionário

O questionário revela-se a par dos dados laboratoriais recolhidos na posição 2 e 4 e posteriormente no laboratório CISA, como uma das partes mais importantes do processo de inquérito em termos de informação recolhida. Uma vez que todos os Trabalhador de Campo, Técnico de Laboratório e Técnico de Enfermagem irão colaborar no seu preenchimento deverão ter em atenção o já expresso no ponto b no capítulo 1.5 e recordar que apenas a mãe ou a responsável pelas crianças com 15 anos ou mais poderão responder às perguntas.

Assegure-se sempre de:

- Utilizar uma linguagem simples, que a inquirida entenda. As diferenças de escolaridade entre o entrevistador e o respondente NÃO PODEM afectar a qualidade dos dados recolhidos.
- Deixe a inquirida tomar o tempo de que necessita para lhe responder.
- Não escreva nenhuma resposta antes de ter a certeza que entendeu a resposta que lhe foi dada.
- No fim, reveja as respostas que acabou de preencher. Pode estar a faltar alguma informação e o melhor é completar o mais cedo possível.

Para além disso deverão saber identificar as partes que lhes dizem respeito e preenche-las na sua totalidade, com letras maiúsculas e assinalando de forma clara a opção respondida com uma cruz (X) ou um visto (√), sem exceder os espaços reservados para essa linha. Deste modo, a distribuição das responsabilidades é:

Trabalhador de Campo fixo na posição 1: deverá preencher os espaços vazios em negrito nas questão 1 e 2
Para além disso deve preencher o cabeçalho, bem como a ID do agregado familiar
Técnico de Laboratório na posição 2: deverão preencher todas as questões do quadro 8

Coordenador na posição 3: deverá preencher as questões 6 a 10.1 no **quadro 1**, referentes à Mãe/responsável das crianças, as questões 5 e 6 do **quadro 2** (referentes às crianças inquiridas nesse agregado familiar apenas! e não a todas as crianças do agregado familiar)
Deve ainda responder a todas as questões dos **quadros 3, 4 e 5**. Estas questões estão relacionadas com a malária, schistossomiase e parasitoses intestinais respectivamente, e a Mãe deverá

responder por ela e pelas crianças. Deverá seguir a ordem das perguntas, dando tempo para a resposta da mãe.

Técnico de Enfermagem na posição 4: Deverá iniciar o registo pelo peso, altura e PMBA nos espaços sombreados (questões 4 e 5 no **quadro 1** e 8 a 10 no **quadro 2**):

1. Informação da Mãe (Demográfica e Peso/altura)		
1. IDPerm:		2. Nome Completo:
3. Data nascimento: / /	4. Altura (cm):	5. Peso (kg):

8. Altura: _________ cm	9. Peso: _________ Kg	10. PBMA (criança >1e <5): ________ cm

Deverá ainda preencher todas as questões dos **quadros 6 e 7**.

No **quadro 6**, ter em atenção que apenas deverá preencher a questão 7 (relativa à vacinação) se houver um cartão de saúde como prova dessas tomas.

No **quadro 7**, o preenchimento da toma dos medicamentos deverá ser efectuado imediatamente a seguir à sua administração de modo a não ocorrerem omissões ou trocas. Mesmo no caso em que não há administração de medicamentos tal deve ser registado.

7. Controlo de administração de medicamentos e encaminhamento de casos – A preencher pela Enfermeira

	Mãe	Criança#1	Criança#2	Criança#3	Criança#4
1. Tomou algum medicamento para a malária na última semana? (**Nota: Se sim,** não administrar Coartem)	S □ N □ NS □	S □ N □ NS □	S □ N □ NS □	S □ N □ NS □	S □ N □ NS □
2. Administração de Albendazol	Sim □ Não □	Sim □ Não □	Sim □ Não □	Sim □ Não □	Sim □ Não □
3. Administração de Prazinquantel	Sim □ Não □	Sim □ Não □	Sim □ Não □	Sim □ Não □	Sim □ Não □
4. Administração de Coartem	Sim □ Não □	Sim □ Não □	Sim □ Não □	Sim □ Não □	Sim □ Não □
5. Administração de Quinino	Sim □ Não □	**Não Aplicável**			
7. Administração de SP/Fansidar (TIP)	Sim □ Não □				
8. Encaminhamento de Anemia [1]	Sim □ Não □	Sim □ Não □	Sim □ Não □	Sim □ Não □	Sim □ Não □
9. Encaminhamento de Malária[2]	Sim □ Não □	Sim □ Não □	Sim □ Não □	Sim □ Não □	Sim □ Não □
10. Encaminhamento de Malnutrição [3]	Sim □ Não □	Sim □ Não □	Sim □ Não □	Sim □ Não □	Sim □ Não □
11. Entrega de 1 mosquiteiro	Sim □ Não □ Porque: ___________________________				

7. Questões Comuns e Resolução de Problemas

Qual o 1º passo quando se chega ao bairro para fazer a recolha de dados?

Ao chegar ao bairro, e durante o processo de montagem das posições 1 a 5, o Trabalhador de Campo móvel da 1ª posição e o supervisor de campo vão verificar as pessoas presentes e a existência de fezes, e organizá-las numa fila, sendo que:

1- Dar prioridade aos agregados familiares completos com as amostras de fezes;

2- Motivar os agregados familiares a recolher a amostra de fezes caso estejam presentes mas sem a amostra;
3- Dar colectores alternativos de fezes a quem não os tem;
4- Conferir os presentes com a Listagem de agregados familiares e membros elegíveis e seleccionados na coluna respectiva (dia 1 ou 2 do inquérito), assinalando com um "visto" (√). Deve ter recebido esta listagem com as anotações do Trabalhador de Campo avançado (poderá ver se ele conseguiu no dia anterior avisar todos os agregados familiares ou quais os em falta).

Se após a 1ª verificação não tiverem presentes todos os agregados familiares esperados o que fazer?

O Trabalhador de Campo móvel (com motorista, se necessário) após esta verificação vai a casa das pessoas ver se elas estão enquanto o Trabalhador de Campo fixo vai adiantando o processo das famílias presentes.

Se houver falta de participantes, o que fazemos?

Chamar residentes de agregados familiares suplentes que completem o critério de uma mãe e de uma criança, até completar o número de participantes necessários – coordenar com supervisor (a idade de cada inquirido é determinada pela sua data de nascimento, ver anexo).

Se houver novos agregados familiares elegíveis no bairro, o que fazer?

Aguardam pelo fim do estudo dos programados, e se faltarem números de inquiridos, são recrutados no fim do dia, sendo estes registados na listagem de novos agregados familiares/membros elegiveis – após este registo preenchem-se todos os formulários usuais. No cabeçalho, para atribuição do ID do agregado familiar e ID do membro – assegura-se que se escolhe um nmr de agregado familiar maior que o último registado pelo SVD (consultando tabela bairros seleccionados). (por exemplo, no caso de Kinguxi, em que há 337 agregados familiares dar-se-ía o ID KGX-00-338 e ID de 1 a 5 aos residentes que participassem no estudo)

Se os membros seleccionados dos agregados familiares seleccionados aparecerem, mas não tenham com eles as amostras de fezes?

Se não houver fezes do dia anterior entrega-se um novo colector – um saco ou copo de plástico – mas primeiro certifica-se o que aconteceu ao anterior e pede-se para voltar no dia seguinte.

A ideia é recolher amostras de fezes, urina e sangue de cada participante mas poderá haver casos em que uma das amostras esteja ausente.
