# Supplementary material for: Schistosomiasis and soil-transmitted helminthiasis preventive chemotherapy: Adverse events in children from 2 to 15 years in Bengo province, Angola
Source: PLoS One. 2020 Mar 11;15(3):e0229247. doi: 10.1371/journal.pone.0229247 (PMC7065752; doi:10.1371/journal.pone.0229247)
Supplement: Attachment — Submitted filename: AE_NTDs - Response to Reviewers Jan20.docx [file pone.0229247.s003.docx]

Editorial Board

# *PLOS ONE*

10^th^ January 2020

Dear Editor,

Please find enclosed the revised manuscript “Schistosomiasis and soil-transmitted helminthiasis preventive chemotherapy: adverse events in children from 2 to 15 years in Bengo Province, Angola” in two versions: a marked-up copy and an unmarked version.

We wish to thank this opportunity, carful revision and adequate comments that improve the final manuscript, as you may see on the enclosed documents. Further detailed answers to the reviewers are stated bellow.

Regarding the journal requirements, a full revision was made and we hope that everything is correct. In relation to data availability, we confirm that there is no ethical or legal restriction on sharing a de-identified data set that we will share upon acceptation of this manuscript.

**Response to Reviewers**

| **Review comments to the Author** | **Authors´ response** | **Topic location in manuscript** |
| --- | --- | --- |
| **Reviewer # 1** | | |
| This paper describes and quantifying the adverse affects of Praziquantel treatment in a cohort of people. The findings are important in raising awareness of adverse affects and also to show the level of severity or not. There are very few corrections needed and I have no specific comments. The English could be improved in places so I suggest the authors have a careful read to try and improve the English as needed. | Thanks for the remark.  We revised the text to improve written English. | All text. |
| **Reviewer # 2** | | |
| Schistosomiasis and STH PC: Adverse events in children in Bengo province, Angola.  This ms is fine and adds to the information on adverse events during MDA and control programs in preschool age children and school age children. It looks at PZQ and ALB and concludes that treatment programs should continue despite small amounts of adverse events.  Title: fine Abstract: fine – minor corrections –  L30 maybe put gender in here.  L32 correct spelling - Schistosoma from schistosome  L32 ‘follow-up’ – treatment or questionnaire – clarify  Introduction:  General points – if using acronyms spell out in first use then use the acronym  A clear definition of adverse events is needed; define the difference between mild/severe.  L56 -- ‘60, 10 and 65 thousand’ what? Unsure what is meant DALYs? Deaths?  L58 reference number needed for Figueiredo et al  L62 using geohelminth here – define.  L65 can used PC common acronym for preventative chemotherapy  L67 may be use mass drug administration (MDA) instead of targeted unless you are performing test and treat control strategy?  L70 use a ] instead of ) to enclose citation.  L74 consider word change to ‘normally’ from ‘possibly’ | Thanks for the remark. We seek to introduce the clarifications and corrections indicated.    L30: included the gender  L32: alterations done  We clarified the general aspects pointed out.  L56: Refers to deaths.  L58: Reference introduced  L62: We exchanged “geohelminth” exchange for “STH”  L65: done  L67: change done  L70: done  L74: change done. | L30 - L74 |
| Methods:  Map?  L112 odd phrasing – ‘selected by convenience’?  L121 odd phrasing – ‘two deworming moments’ ?  L162 reference for the software  Ethical approval – is there an associated number for this study from the ethical committee?  It might be good to have the number of children enrolled in the study in your methods? | Map: thank you for the suggestion, but we opted for not include a map of the area to not overload the manuscript  L112: we removed "by convenience"  L121: we removed "two deworming moments"  L162: correction done.  We enter the number of children enrolled in the methods. | L112 – L162 |
| Results:  All tables – change ‘yes’ ‘no’ to egg positive negative or something. These need to be made clearer – particularly table 2 – it contains a lot of information but perhaps consider a graph or different way to display which highlights important results.  L185 change ‘themself’ to themselves and ‘caretakers’ to guardians  L188 Plasmodium in full not just P. | We introduced suggested changes.  Table 2 shows only the number (%) of participants (PSAC and SAC) who were surveyed (1st line of numbers) and those who reported the various types of AEs in both surveys.  L185: Alterations done  L188: done | L185 –L188 |
| Discussion:  L259 – 262 Reword and break up – strange wording here.  L269 italicize S. haematobium  L273 ref in list please  L280 please clarify – not sure what it means | L259-262: We introduced the suggested rewording.  L269: done  L273: done  L280: Correction done. | L259-280 |
| References:  Check formatting with the journal | Formatting verified. |  |
| **Reviewer #3** | | |
| This is an interesting report on adverse events during systematic preventive administration of praziquantel and albendazole in children. The prevalence in this area in Angola is impressive and thus the study area is adapted to have excellent data on the subject.  The paper is well written although the methodology and result section lack on clarity and can not be clear enough without the reading of the complementary documents : this should be improved.  There are some sentences that are unclear because of english or missing items : line 53 to 56 ; line 63 ; 70; 233 ; line 243-246.  In the discussion again it is not clear why the 45 % of uninfected children who have AES should be underdiagnosed ? why it is not possible to have AES with the drug alone ? (line 308)  These different points should be improved. | Thanks for the remark.  We seek to introduce the clarifications and corrections indicated.  L53 – 56: clarification done  L63: clarification done  L70: clarification done  L233: clarification done  L234-246: Correction done.  L308: Correction done. | L53 - 308 |

We look forward to hearing from you at your earliest convenience.

Yours sincerely,

João M. Pedro
